# WTAP regulates postnatal development of brown adipose tissue by stabilizing METTL3 in mice

**DOI:** 10.1093/lifemeta/loac028

**Published:** 2022-10-10

**Authors:** Yuqin Wang, Xinzhi Li, Cenxi Liu, Liying Zhou, Lei Shi, Zhiguo Zhang, Long Chen, Ming Gao, Lanyue Gao, Yuanyuan Xu, He Huang, Jin Li, Zheng Chen

**Affiliations:** HIT Center for Life Sciences, School of Life Science and Technology, Harbin Institute of Technology, Harbin, Heilongjiang 150001, China; HIT Center for Life Sciences, School of Life Science and Technology, Harbin Institute of Technology, Harbin, Heilongjiang 150001, China; School of Life Science, State Key Laboratory of Genetic Engineering, Fudan University, Shanghai 200438, China; Shanghai Key Laboratory of Metabolic Remodeling and Health, Institute of Metabolism and Integrative Biology, Fudan University, Shanghai 200438, China; Department of Cardiology, The First Hospital of Jilin University, Changchun, Jilin 130021, China; Department of Cardiology, The First Hospital of Jilin University, Changchun, Jilin 130021, China; HIT Center for Life Sciences, School of Life Science and Technology, Harbin Institute of Technology, Harbin, Heilongjiang 150001, China; HIT Center for Life Sciences, School of Life Science and Technology, Harbin Institute of Technology, Harbin, Heilongjiang 150001, China; The Key Laboratory of Liaoning Province on Toxic and Biological Effects of Arsenic, School of Public Health, China Medical University, Shenyang, Liaoning 110122, China; The Key Laboratory of Liaoning Province on Toxic and Biological Effects of Arsenic, School of Public Health, China Medical University, Shenyang, Liaoning 110122, China; Shanghai Key Laboratory of Metabolic Remodeling and Health, Institute of Metabolism and Integrative Biology, Fudan University, Shanghai 200438, China; School of Life Science, State Key Laboratory of Genetic Engineering, Fudan University, Shanghai 200438, China; HIT Center for Life Sciences, School of Life Science and Technology, Harbin Institute of Technology, Harbin, Heilongjiang 150001, China

**Keywords:** WTAP, BAT, m^6^A, postnatal development, METTL3, PRDM16

## Abstract

Brown adipocyte maturation during postnatal development is essential for brown adipose tissue (BAT) to protect animals against cold. Impaired maturation of brown adipocytes leads to cold intolerance. However, the molecular mechanisms that determine the maturation of brown adipocytes during postnatal development are not fully understood. Here, we identify Wilms’ tumor 1-associating protein (WTAP) as an essential regulator in the postnatal development and maturation of BAT. BAT-specific knockout of *Wtap* (*Wtap*-BKO) severely impairs maturation of BAT *in vivo* by decreasing the expression of BAT-selective genes, leading to the whitening of interscapular BAT (iBAT). Single nucleus RNA-sequencing analysis shows the dynamic changes of cell heterogeneity in iBAT of *Wtap*-BKO mice. Adult mice with WTAP deficiency in BAT display hypothermic and succumb to acute cold challenge. Mechanistically, WTAP deficiency decreases m^6^A mRNA modification by reducing the protein stability of METTL3. BAT-specific overexpression of *Mettl3* partially rescues the phenotypes observed in *Wtap*-BKO mice. These data demonstrate that WTAP/METTL3 plays an essential role in iBAT postnatal development and thermogenesis.

## Introduction

Interscapular brown adipose tissue (iBAT) is a major tissue for non-shivering thermogenesis through uncoupling protein 1 (UCP1)-dependent respiration [1, 2]. For rodents, brown adipocytes undergo postnatal maturation and then gain full function [3–5]. Impaired maturation of brown adipocytes leads to cold intolerance in adults [5]. In the past two decades, several transcriptional factors such as PR domain-containing protein 16 (PRDM16) and peroxisome proliferator-activated receptor gamma (PPARγ) have been identified as key regulators of brown fat differentiation [4, 6–8]. However, whether RNA processing, such as *N*^6^-methyladenosine (m^6^A) mRNA modification, regulates iBAT development and thermogenesis is not fully understood.

RNA processing including m^6^A mRNA modification is mediated by RNA binding proteins. The key components in m^6^A methyltransferase complex include METTL3, METTL14, and Wilms’ tumor 1-associating protein (WTAP) [9, 10]. METTL3, the key m^6^A methyltransferase, has been shown to regulate early embryonic development [11], neurogenesis [12], diabetes [13], and nonalcoholic steatohepatitis (NASH) [14]. WTAP interacts with METTL3 and METTL14 in the nucleus and serves as a regulatory protein of m^6^A mRNA modification. WTAP has been shown to regulate X chromosome imprinting [15], cell proliferation [16], white adipogenesis [17], and tumorgenesis [18–21] by modulating RNA alternative splicing. Recently, WTAP in the liver has been shown to regulate lipoatrophy and NASH by binding to specific DNA motifs [22]. Both *Mettl3* and *Wtap* knockout mice were embryonic death [11, 23], indicating their importance in embryonic development. We recently reported that METTL3 is an essential regulator for postnatal development of iBAT [5]. However, whether WTAP-mediated RNA processing regulates the maturation of iBAT is largely unknown, and whether WTAP regulates iBAT development depending on METTL3 is also unknown.

Here, we show that WTAP is an essential regulator in the postnatal development of iBAT. BAT-specific deletion of *Wtap* severely impairs the maturation of BAT *in vivo* by decreasing the expression of BAT-selective genes, leading to the whitening of iBAT. Single nucleus RNA-sequencing (snRNA-seq) analysis shows the dynamic changes of cell heterogeneity in iBAT of *Wtap*-BKO mice. Adult mice with WTAP deficiency in BAT display hypothermic and succumb to acute cold challenge. WTAP deficiency decreases m^6^A mRNA modification by reducing the protein stability of METTL3. BAT-specific overexpression of *Mettl3* partially rescues the phenotypes in BAT-specific *Wtap* knockout mice (*Wtap*-BKO). These data reveal a mechanism in which WTAP plays an essential role in iBAT postnatal development and thermogenesis.

## Results

### WTAP, a BAT-enriched and development-associated protein, is essential for the postnatal development of iBAT

**Figure 1:**
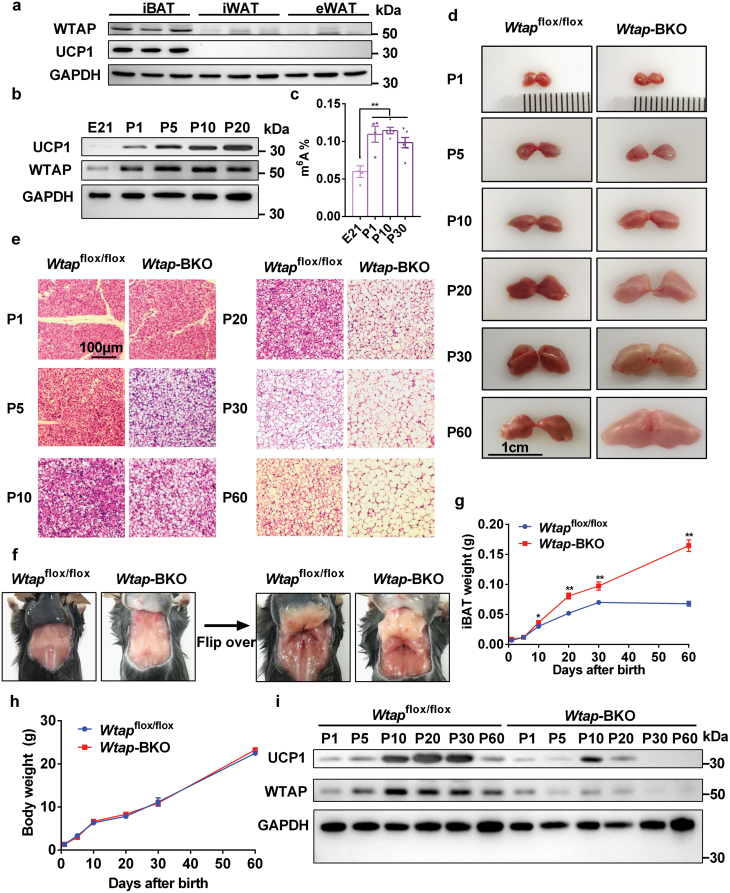
WTAP is essential for the postnatal development of iBAT. (a) WTAP and UCP1 protein levels in iBAT, iWAT, and eWAT of C57BL/6 male mice at the age of 8 weeks were measured by immunoblotting. (b) WTAP and UCP1 protein levels in iBAT at embryonic day 21 (E21) and postnatal days 1, 5, 10, and 20 were measured by immunoblotting. (c) m^6^A levels of total RNA in iBAT at E21, P1, P10, and P30 (*n* = 4–6). (d) Gross appearance of iBATs in *Wtap*^flox/flox^ and *Wtap*-BKO mice at days 1, 5, 10, 20, 30, and 60 after birth. (e) Haematoxylin and eosin (H&E) staining of iBATs in *Wtap*^flox/flox^ and *Wtap*-BKO mice at days 1, 5, 10, 20, 30, and 60 after birth. (f) Gross appearance of iBATs in *Wtap*^flox/flox^ and *Wtap*-BKO mice at the age of 8 weeks. (g and h) iBAT and body weight of *Wtap*^flox/flox^ and *Wtap*-BKO mice at days 1, 5, 10, 20, 30, and 60 after birth (*n* = 3–8). (i) WTAP and UCP1 protein levels in iBAT of *Wtap*^flox/flox^ and *Wtap*-BKO mice at days 1, 5, 10, 20, 30, and 60 after birth. Immunoblotting experiments were repeated three times independently with similar results. *P < 0.05. **P< 0.01. Data represent the mean ± SEM. *n* was the number of biologically independent mice.

BAT-enriched genes may regulate BAT function. Published microarray data (GSE8044) show that WTAP is one of BAT-enriched genes [6]. We also observed that WTAP protein levels were significantly higher in iBAT compared with that in inguinal white adipose tissue (iWAT) and epididymal white adipose tissue (eWAT) (Fig. 1a). To further test whether WTAP is associated with the postnatal development of iBAT, we determined WTAP protein levels in iBAT at different ages after birth. WTAP protein levels were significantly increased in iBAT at 1 day of age, and reached the maximal level at 10 days of age (Fig. 1b). The UCP1 protein levels were also showed a similar expression pattern with WTAP (Fig. 1b) and METTL3 [5]. m^6^A levels in total RNA were also significantly increased at 1 day of age and remained at similar levels at 10 and 30 days of age (Fig. 1c). These data demonstrate that WTAP is selectively expressed in iBAT, and WTAP/METTL3/m^6^A are upregulated in iBAT during postnatal development, indicating that WTAP may play an important role in the postnatal development of iBAT.

To further determine whether WTAP regulates iBAT postnatal development, we need to generate *Wtap*-BKO mice. *Ucp*1-Cre transgenic mice were widely used to generate BAT-specific knockout mice. However, a recent publication showed that Cre recombinase in *Ucp*1-Cre mice was expressed not only in BAT and iWAT, but also in the kidney and hypothalamus [24], indicating the *Ucp*1-Cre mice are not good enough to generate BAT-specific knockout mice. To avoid this non-specificity of *Ucp*1-Cre, we tested *Ucp1*-iCre mice, in which IRES-Cre was inserted between the exon 6 and the 3ʹ-UTR to allow UCP1 and iCRE expression at the same time with lower levels [25]. We generated *Wtap*-BKO mice by crossing *Wtap*-floxed mice (Supplementary Fig. S1a and b) with *Ucp1*-iCre transgenic mice. *Ucp1*-iCre has been shown to delete genes in iBAT at 5 days of age [5]. To test the specificity of the deletion in BAT, we performed immunoblotting in different tissues. As shown in Supplementary Fig. S1c, WTAP was specifically deleted in iBAT but not in other tissues, such as liver and brain in adult *Wtap*-BKO mice. We also noted that WTAP was highly enriched in iBAT, liver, and brain, compared with that in eWAT and skeletal muscle (Supplementary Fig. S1c). We did not observe any difference in body weight (Supplementary Fig. S1d), iBAT morphology (Supplementary Fig. S1e), iBAT weight (Supplementary Fig. S1f), or cold challenge (Supplementary Fig. S1g) between *Wtap*^flox/flox^ and *Ucp1*-iCre mice. Therefore, we used *Wtap*^flox/flox^ mice as the control for *Wtap*-BKO mice in the following experiments. Surprisingly, the morphology of iBAT in *Wtap*-BKO mice appeared abnormal, enlarged, and “whitening” roughly after 10 days of age (Fig. 1d–f). Consistently, the weight of iBAT in *Wtap*-BKO mice was significantly higher than that of *Wtap*^flox/flox^ mice (Fig. 1g), which did not significantly affect the body weight of *Wtap*-BKO mice during postnatal development (Fig. 1h). The enlarged iBAT was attributable to large cytosolic lipid droplet accumulation, resulting in an increase in average adipocyte size (steatotic hypertrophy) (Fig. 1e). The key thermogenic protein, UCP1, was significantly increased during postnatal development in iBAT of *Wtap*^flox/flox^ mice, whereas its expression was much less in iBAT of *Wtap*-BKO mice (Fig. 1i). Consistently, WTAP protein levels in iBAT of *Wtap*-BKO mice began to decline at 5 days of age (Fig. 1i). These data indicate that WTAP is necessary for postnatal development of iBAT.

### BAT-specific deletion of *Wtap* results in decreased energy expenditure, which does not promote HFD-induced obesity

**Figure 2:**
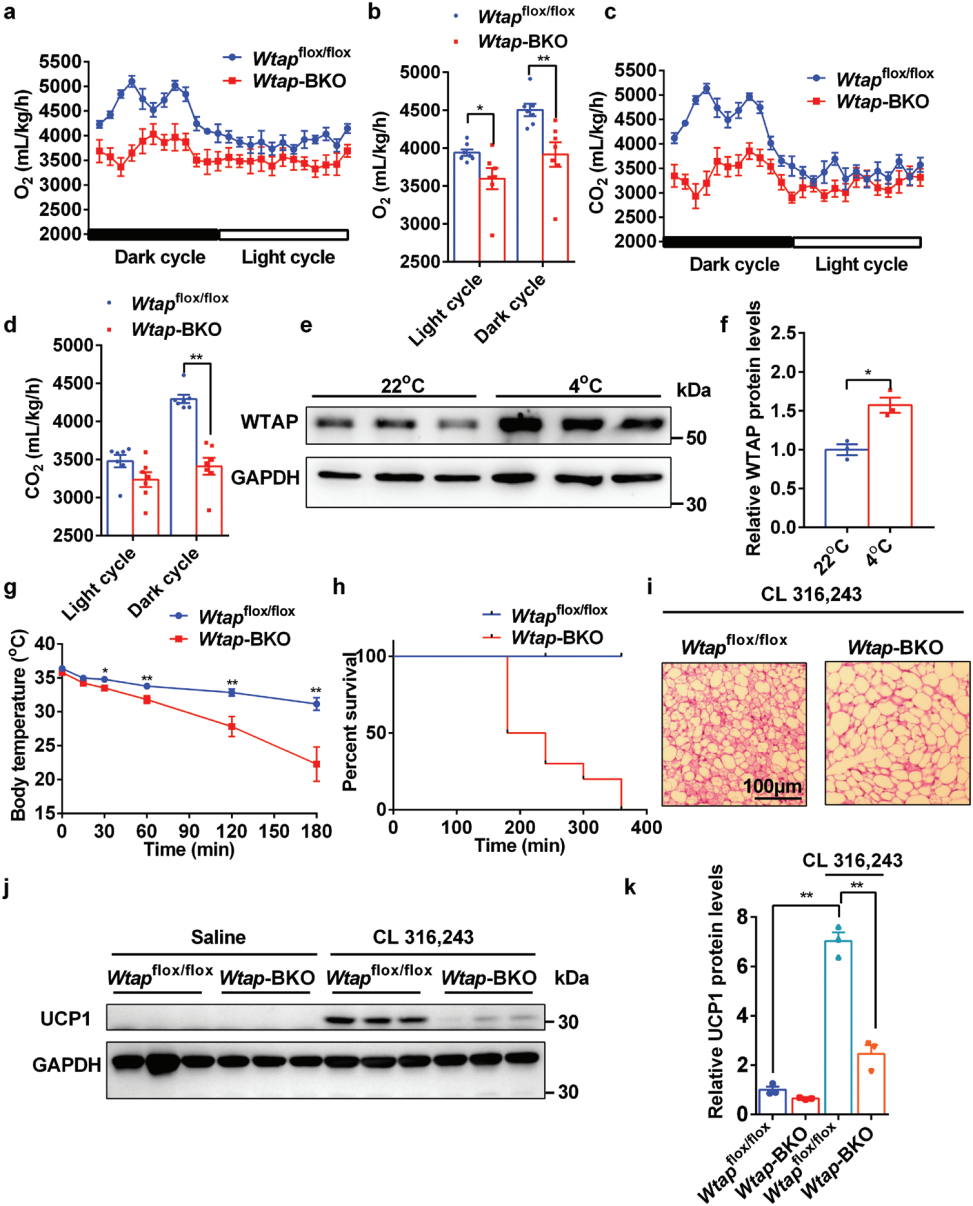
*Wtap* deficiency in BAT causes decreased energy expenditure, cold intolerance, and impaired browning of iWAT. (a and b) The O_2_ consumption rates in 10-week-old *Wtap*^flox/flox^ and *Wtap*-BKO mice at room temperature (*n* = 7). (c and d) The CO_2_ production rates in 10-week-old *Wtap*^flox/flox^ and *Wtap*-BKO mice at room temperature (*n* = 7). (e and f) The WTAP protein levels in iBATs of WT mice treated with or without acute cold exposure for 6 h (*n* = 3). (g) The rectal temperature of 8-week-old *Wtap*^flox/flox^ and *Wtap*-BKO mice during acute cold exposure (4°C) (*n* = 10). (h) Percent survival of 8-week-old *Wtap*^flox/flox^ and *Wtap*-BKO mice during acute cold exposure (4°C) (*n* = 10). (i–k) *Wtap*^flox/flox^ and *Wtap*-BKO mice were injected with CL 316,243 for 4 days to induce browning of WAT. H&E staining of iWAT in *Wtap*^flox/flox^ and *Wtap*-BKO mice after CL 316,243 treatment was shown (i). UCP1 protein levels in iWAT of *Wtap*^flox/flox^ and *Wtap*-BKO mice treated with or without CL 316,243 were measured by immunoblotting (j and k) (*n* = 3). *P < 0.05. **P < 0.01. Data represent the mean ± SEM. *n* was the number of biologically independent mice.

Impaired postnatal development of iBAT leads to decreased energy expenditure [5]. Expectedly, *Wtap*-BKO mice displayed lower oxygen (O_2_) consumption and CO_2_ production rates during both light and dark cycles (Fig. 2a–d) with similar amounts of food intake compared with *Wtap*^flox/flox^ controls (Supplementary Fig. S2a). Surprisingly, *Wtap*-BKO mice showed increased physical activity in daytime but similar amounts of physical activity in nighttime compared with *Wtap*^flox/flox^ controls (Supplementary Fig. S2b). Physical activity may be associated with skeletal muscle function. The running time to exhaustion was significantly increased in *Wtap*-BKO mice (Supplementary Fig. S2c), indicating that *Wtap*-BKO increases skeletal muscle function, which contributes to the increased physical activity.

Next, we tested the need for WTAP in thermogenesis *in vivo*. WTAP protein levels were significantly increased after acute cold exposure (4°C for 6 h) (Fig. 2e and f), indicating that WTAP may promote thermogenesis. Strikingly, *Wtap*-BKO mice displayed a rapid loss of body temperature, leading to mouse death within 3–6 h after cold exposure, whereas all *Wtap*^flox/flox^ control mice maintained their body temperature and survived (Fig. 2g and h). These data suggest that WTAP in BAT is essential for thermogenesis.

WTAP is exclusively expressed in iBAT, and BAT-specific deletion of *Wtap* impairs acute cold-induced thermogenesis, indicating that WTAP may regulate the browning of WAT in response to the β-adrenergic agonist. To further test this hypothesis, *Wtap*-BKO mice and *Wtap*^flox/flox^ controls were injected with CL 316,243 for 4 days to induce browning of WAT. As shown in Fig. 2i–k, multiple injections of CL 316,243-induced browning of iWAT and higher expression of UCP1 in *Wtap*^flox/flox^ mice, whereas the induction of iWAT browning and the UCP1 expression were significantly abolished in *Wtap*-BKO mice. These data demonstrate that BAT-specific deletion of *Wtap* impairs the browning of WAT in response to the β-adrenergic agonist.

Reduced energy expenditure contributes to obesity in both rodents and humans [26, 27]. To determine whether *Wtap*-BKO mice are sensitive to high-fat diet (HFD)-induced obesity, *Wtap*-BKO and *Wtap*^flox/flox^ control mice were fed with HFD, and body weight was measured weekly. As shown in Supplementary Fig. S2d, *Wtap*-BKO and *Wtap*^flox/flox^ mice gained similar body weight after feeding with HFD. Consistent with the previous observation, the iBAT weight was significantly higher in *Wtap*-BKO mice (Supplementary Fig. S2e), and the morphology of iBAT in *Wtap*-BKO mice also appeared abnormal, enlarged, and “whitening” after HFD-feeding (Supplementary Fig. S2f). We also measured systemic glucose homeostasis and insulin resistance on mice fed with HFD for 13 weeks (21 weeks old). As shown in Supplementary Fig. S2g and h, *Wtap*-BKO mice displayed similar glucose intolerance and insulin resistance with *Wtap*^flox/flox^ mice. Insulin-induced p-AKT(S473) levels were also similar in the livers of *Wtap*-BKO and *Wtap*^flox/flox^ mice (Supplementary Fig. S2i). These data suggest that decreased expression of *Wtap* in iBAT does not affect HFD-induced obesity and metabolic syndrome, which is likely due to the increased physical activity (Supplementary Fig. S2b).

### BAT-specific deletion of *Wtap* largely changes gene expression profile in iBAT

**Figure 3:**
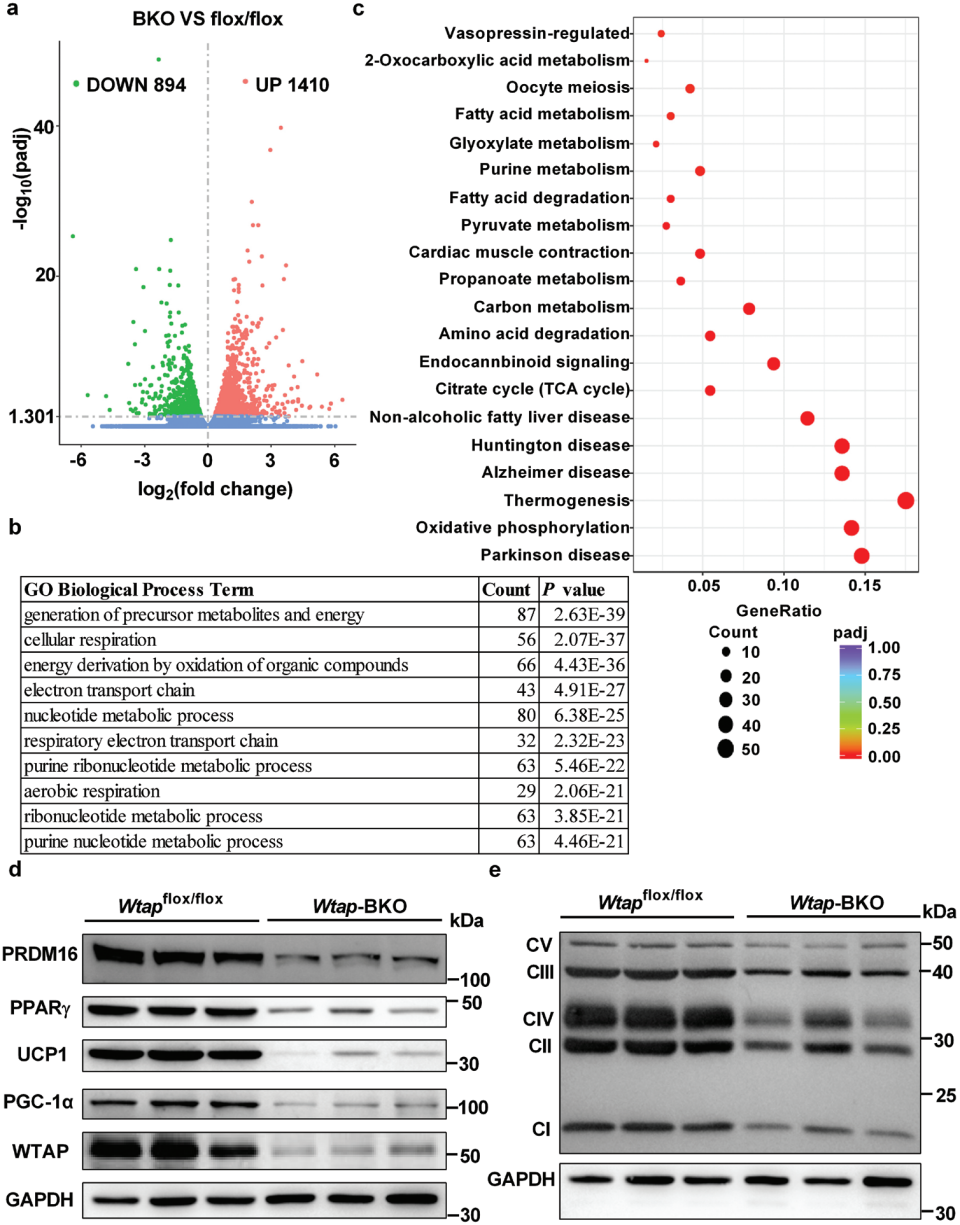
BAT-specific deletion of *Wtap* dramatically decreases expression of BAT-selective genes. RNA-seq analysis was performed in the iBATs of *Wtap*^flox/flox^ and *Wtap*-BKO mice of 8 weeks old. (a) The differentially expressed genes (BKO vs. flox/flox) including 894 downregulated genes and 1410 upregulated genes were illustrated in a volcanoplot (|log2FoldChange|>0 and *P* value < 0.05) (*n* = 3). (b) Top GO biological process terms enriched in downregulated genes. (c) KEGG analysis of the downregulated genes. (d) PRDM16, PPARγ, UCP1, PGC-1α, WTAP, and GAPDH protein levels in iBATs of 8-week-old *Wtap*^flox/flox^ and *Wtap*-BKO mice were determined by immunoblotting (*n* = 3). (e) Mitochondrial complex protein levels in iBATs of 8-week-old *Wtap*^flox/flox^ and *Wtap*-BKO mice were determined by immunoblotting (*n* = 3). *n* was the number of biologically independent mice.

To further explore the molecular mechanisms of the impaired postnatal development of iBAT in *Wtap*-BKO mice, we examined the whole transcriptional profiles of iBAT in both *Wtap*-BKO and *Wtap*^flox/flox^ mice by performing RNA-seq analysis. As shown in Fig. 3a, 894 genes were downregulated, whereas 1410 genes were upregulated. Gene Ontology (GO) analysis showed that the downregulated genes were related to the generation of precursor metabolites and energy, cellular respiration, energy derivation by oxidation of organic compounds, electron transport chain, nucleotide metabolic process, and purine ribonucleotide metabolic process (Fig. 3b). KEGG pathway analysis showed that the downregulated genes were associated with oxidative phosphorylation, thermogenesis, TCA cycle, carbon metabolism, and fatty acid metabolism signaling pathways (Fig. 3c). We then performed metabolomics analysis. As shown in Supplementary Fig. S3a and b, metabolites involved in TCA cycle did not change, but acetyl-CoA and succinyl-CoA were downregulated, which indicates that the energy generation from some branch chain amino acids is decreased. The downregulated AMP and guanosine (Supplementary Fig. S3c) indicate that the purine de novo synthesis via pentose phosphate pathway is decreased in iBAT of *Wtap*-BKO mice, which is consistent with RNA-seq data. Furthermore, PRDM16, PPARγ, UCP1, PGC-1α, and WTAP protein levels were dramatically reduced in iBAT of *Wtap*-BKO mice (Fig. 3d). In addition, most of the genes related to mitochondrial oxidative phosphorylation (OXPHOS), including the components of complexes I, II, III, IV, and V, were dramatically reduced in *Wtap*-BKO mice (Fig. 3e). These data suggest that BAT-specific deletion of *Wtap* impairs postnatal development of iBAT by decreasing expression of BAT-selective genes.

### BAT-specific deletion of *Wtap* largely changes cellular composition and characteristics of various cell types in iBAT

**Figure 4:**
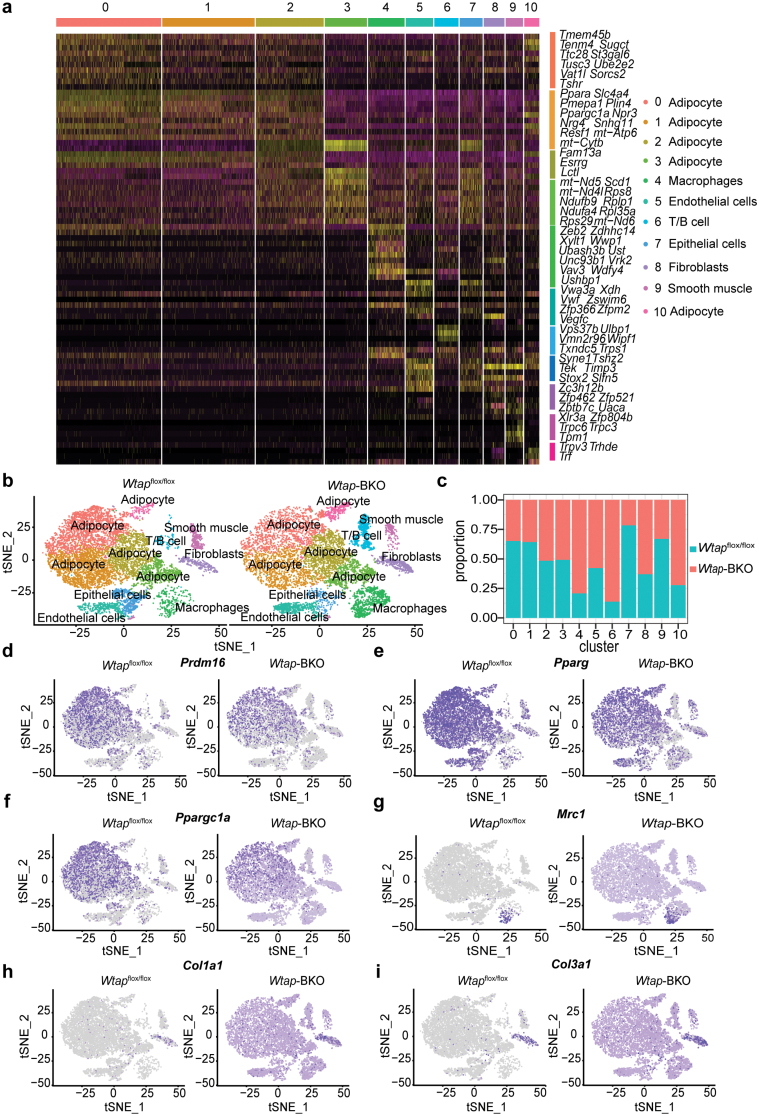
BAT-specific deletion of *Wtap* largely changes cell heterogeneity in iBAT. Cell nuclei were isolated from iBAT in *Wtap*^flox/flox^ and *Wtap*-BKO mice of 8 weeks old, and snRNA-seq analysis was performed. (a) Heatmap showing normalized expression levels of marker genes helping to identify eleven cell types present in the snRNA-seq data from iBAT. (b) Integrated analysis of snRNA-seq data from iBAT in *Wtap*^flox/flox^ and *Wtap*-BKO mice at the age of 8 weeks. (c) Percent contribution of iBAT cells from *Wtap*^flox/flox^ (blue) and *Wtap*-BKO (red) mouse in each cluster. (d–i) Distribution of the expression of *Prdm16* (d), *Pparg* (e), *Ppargc1α* (f), *Mrc1* (g), *Col1a1*(h), and *Col3a1* (i) within tSNE plot in iBAT of *Wtap*^flox/flox^ and *Wtap*-BKO mice.

Not all brown adipocytes are equal. Cell heterogeneity is dynamically changed in iBAT under different conditions [28]. To elucidate cell heterogeneity and their dynamic changes in iBAT of *Wtap*-BKO and *Wtap*^flox/flox^ mice, we performed snRNA-seq on iBAT. In total, 8500–9500 unique cell nuclei were sequenced and analyzed. Cells were classified into several distinct clusters, most of which were easily identifiable by defined marker expression, including adipocytes, macrophages, endothelial cells, T/B lymphocytes, epithelial cells, fibroblasts, and smooth muscle cells (Fig. 4a–c). *Prdm16*, *Pparg*, and *Ppargc1α* mRNA levels were significantly decreased in brown adipocytes of *Wtap*-BKO mice (Fig. 4d–f and Supplementary Fig. S4a–c), which is consistent with the immunoblotting data (Fig. 3d). We also observed remarkably increased proportions for macrophages and T/B lymphocytes in the iBAT of *Wtap*-BKO mice (Fig. 4b and c). In addition, the macrophage-related gene (*Mrc1*) and fibrosis-related genes such as *Col1a1* and *Col3a1* were upregulated in macrophage, fibroblasts, and other cell types (Fig. 4g–i and Supplementary Fig. S4d–f). These data indicate that the cellular composition and characteristics of many cell types in iBAT of *Wtap*-BKO mice are changed, which is likely due to the immaturation of brown adipocytes.

### WTAP controls brown adipogenesis *in vitro*

**Figure 5:**
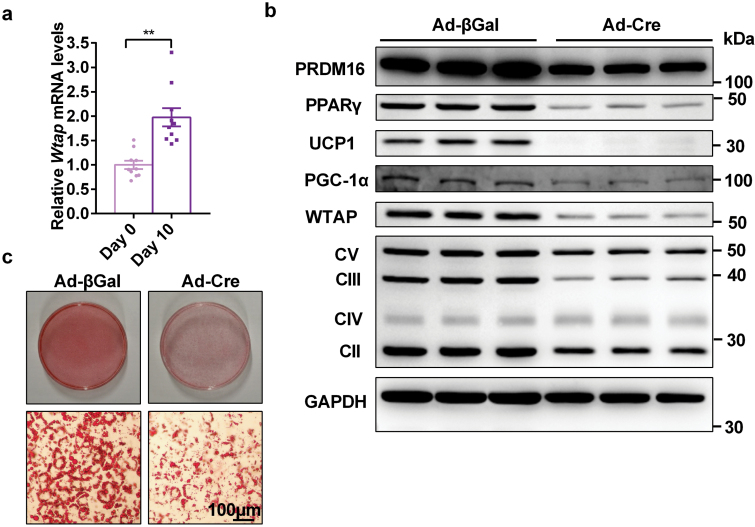
WTAP is essential for brown adipogenesis. (a) The *Wtap* mRNA levels in mature primary brown adipocytes (10 days after differentiation) and preadipocytes (before differentiation) (*n* = 10). (b and c) Brown fat SVF was isolated from 4-week-old *Wtap*^flox/flox^ mice and cultured in a growth medium. 100% confluent cells were infected with Cre- or βGal–expressing adenovirus in a growth medium overnight. The medium was switched to induction medium for 72 h to induce adipogenic differentiation, and then cells were maintained in differentiation medium for 4 days. Cells were then used for immunoblotting analysis (b) and Oil red O staining (c). The cell culture experiments were repeated three times independently with similar results. **P < 0.01. Data represent the mean ± SEM. *n* was the number of biologically independent cell samples.

To determine whether WTAP primarily regulates brown adipogenesis, we performed *in vitro* cell culture experiments. *Wtap* mRNA levels were significantly higher in mature primary brown adipocytes compared with preadipocytes (Fig. 5a), indicating that WTAP may directly regulate the differentiation of brown adipocytes. To test whether WTAP directly regulates the differentiation of brown adipocytes, we harvested the stromal vascular fraction (SVF) from the brown fat pads of *Wtap*^flox/flox^ mice. Primary brown preadipocytes were infected with Ad-βGal and Ad-Cre adenovirus and differentiated to mature brown adipocytes. As expected, Cre adenovirus infection caused the deletion of *Wtap* in primary brown adipocytes (Fig. 5b). Deletion of *Wtap* significantly impaired the differentiation of precursor cells, as revealed by decreased Oil Red O staining (Fig. 5c), the decreased expression of general brown adipocyte markers, such as PRDM16, PPARγ, UCP1, and PGC-1α (Fig. 5b), and the reduced components of mitochondrial complexes II, III, and V (Fig. 5b). These results suggest that WTAP is necessary for brown adipogenesis *in vitro*.

### WTAP is necessary for m^6^A mRNA modification by stabilizing METTL3 in BAT

**Figure 6:**
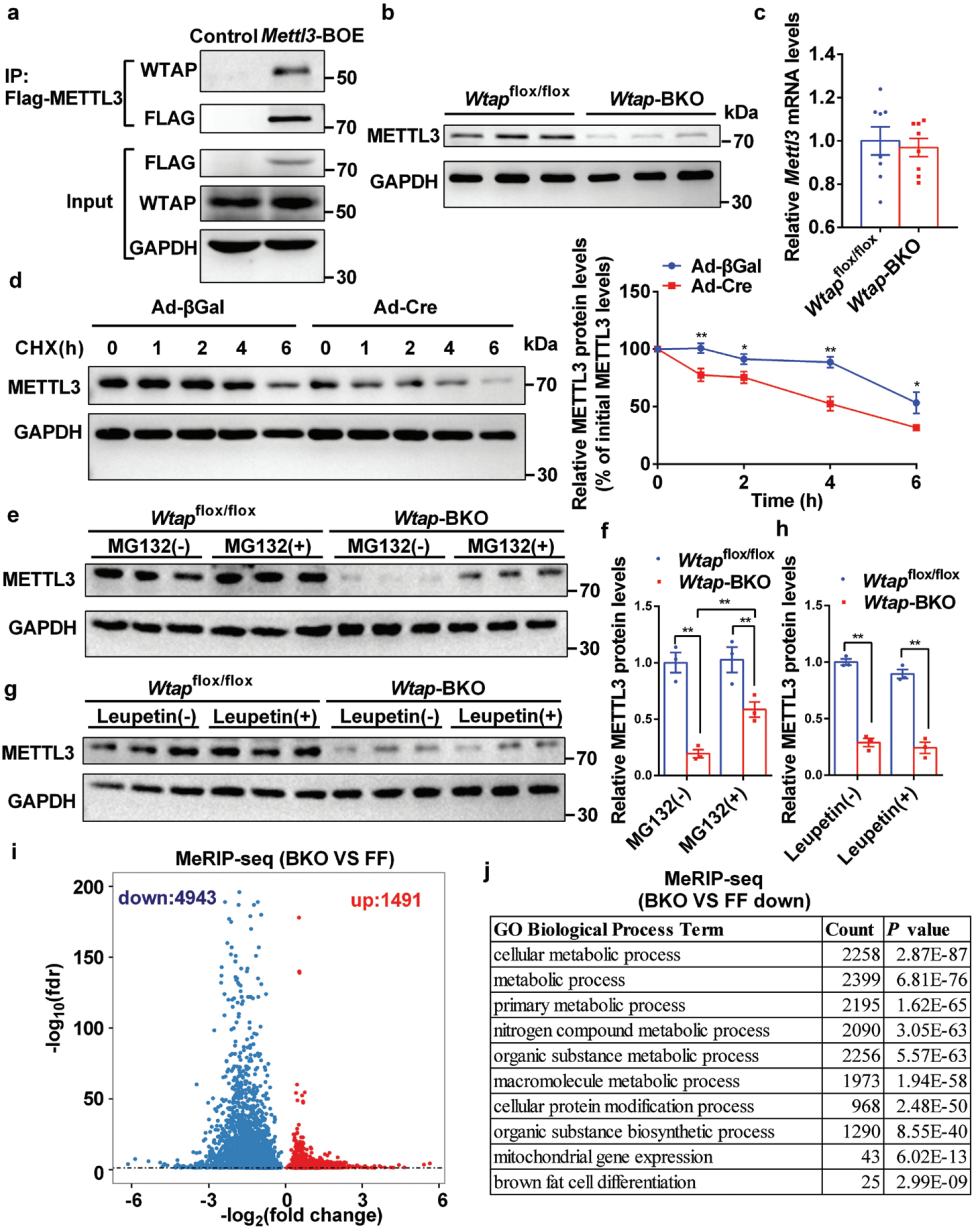
WTAP is essential for m^6^A mRNA modification in iBAT. (a) BAT-specific *Mettl3*-overexpressing (*Mettl3*-BOE) mice were generated by crossing *STOP-Mettl3*^*+/-*^ mice, in which a *STOP-Flag-Mettl3* cassette was inserted in the *Rosa26* allele using the CRISPR-Cas9 technique, with *Ucp1-iCre* transgenic mice. The genotype of the *Mettl3-*BOE mice was *STOP-Mettl3*^*+/-*^ *Ucp1-iCre*^*+/-*^. iBAT extracts from control and *Mettl3*-BOE mice at the age of 8 weeks were immunoprecipitated with Flag beads and then immunoblotted with anti-WTAP antibody. Flag, WTAP, and GAPDH protein levels in the input lysates were determined by immunoblotting. The samples were derived from the same experiment and the blots were processed in parallel. These experiments were repeated three times independently with similar results. (b) METTL3 and GAPDH protein levels in iBATs of 8-week-old *Wtap*^flox/flox^ and *Wtap*-BKO mice were determined by immunoblotting (*n* = 3). (c) The relative *Mettl3* mRNA levels in iBATs of 8-week-old *Wtap*^flox/flox^ and *Wtap*-BKO mice were determined by RT-qPCR (*n* = 8). (d) Brown fat SVF was isolated from 4- to 6-week-old *Wtap*^flox/flox^ mice and cultured in a growth medium. 100% confluent cells were infected with Cre- or βGal–expressing adenovirus in growth medium overnight. The infected cells were switched to an induction medium for 24 h to induce adipogenic differentiation, and then treated with cycloheximide (CHX, 50 ng/mL) for indicated periods. Cells were then harvested for immunoblotting analysis of METTL3 and GAPDH protein levels. The relative METTL3 protein levels were represented as the percentage of the band densities at 0 h. Quantifications of relative METTL3 protein levels were from seven independent experiments. (e and h) The iBAT was dissected from 4- to 6-week-old *Wtap*^flox/flox^ and *Wtap*-BKO mice and cut into 10 mg pieces. Pieces of iBAT were randomly divided into two groups. One group was treated with or without MG132 (100 μmol/L) (e and f), and the other group was treated with or without leupetin (100 μmol/L) (g and h) at 37°C for 6 h. METTL3 and GAPDH protein levels were measured by immunoblotting. Quantifications of relative METTL3 protein levels were from three independent mice. (i and j) The MeRIP-seq analysis of iBATs was performed in 8-week-old *Wtap*^flox/flox^ and *Wtap*-BKO mice. The differently regulated m^6^A peaks-associated genes (BKO vs flox/flox) including 4943 downregulated m^6^A peaks-associated genes and 1491 upregulated m^6^A peaks-associated genes were illustrated in a volcanoplot (log2FoldChange > 0 and *P* value < 0.05) (i). GO biological process enrichment analysis of downregulated m6A peaks-associated genes (j). *n* was the number of biologically independent mice or cell samples.

Next, we wanted to explore the detailed molecular mechanisms in which WTAP regulates the postnatal development of iBAT. It has been shown that WTAP interacts with METTL3 in the nucleus and serves as a regulatory protein of m^6^A mRNA modification [9, 10]. We recently reported that METTL3 is an essential regulator for the postnatal development of iBAT by regulating m^6^A mRNA modification [5]. We further confirmed that WTAP interacted with METTL3 in iBAT (Fig. 6a). BAT-specific deletion of *Wtap* showed decreased METTL3 protein but not its mRNA levels (Fig. 6b and c), indicating that WTAP regulates METTL3 protein stability. We then performed protein stability assays. As shown in Fig. 6d, the deletion of *Wtap* in primary brown adipocytes significantly decreased METTL3 protein stability. Furthermore, MG132, a proteasome inhibitor, was able to increase METTL3 protein levels in the iBAT of *Wtap*-BKO mice (Fig. 6e and f), whereas leupetin, a lysosome inhibitor, was not able to increase METTL3 protein levels (Fig. 6g and h), indicating that *Wtap*-BKO reduces METTL3 protein stability depending on proteasome.

To further determine whether WTAP regulates m^6^A modification of mRNA related to brown fat differentiation and thermogenesis, we performed m^6^A RNA immunoprecipitation sequencing (MeRIP-seq) analysis in iBAT of *Wtap*-BKO and *Wtap*^flox/flox^ control mice. Each sample was pooled from 8 mice for each group. Consistent with published MeRIP-seq results [29], the m^6^A peaks identified in iBAT of *Wtap*^flox/flox^ control mice were enriched at stop codon and 3ʹ-UTR and were characterized by the canonical GGACU motif (Supplementary Fig. S5a and b). However, the enrichment of the m^6^A peaks at stop codon and 3ʹ-UTR were decreased in iBAT of *Wtap*-BKO mice (Supplementary Fig. S5a). In the iBAT of *Wtap*^flox/flox^ mice, we identified about 18,400 significant m^6^A peaks (false discovery rate < 0.05) in about 9732 transcripts (Supplementary Table S1). *Wtap*-BKO mice displayed lower m^6^A levels in iBAT (Fig. 6i). There were 4943 transcripts exhibiting decreased m^6^A levels in the iBAT of *Wtap*-BKO mice (Fig. 6i and Supplementary Table S2). GO analysis showed that genes with downregulated m^6^A peaks were associated with metabolic process, cellular protein modification process, mitochondrial gene expression, and brown fat cell differentiation (Fig. 6j). KEGG analysis showed that genes with downregulated m^6^A peaks were associated with metabolic pathways, insulin signaling pathway, MAPK signaling pathway, AMPK signaling pathway, and protein processing in the endoplasmic reticulum (Supplementary Fig. S6). Importantly, genes with downregulated m^6^A peaks included *Prdm16* and *Pparg* (Fig. 6i and Supplementary Table S2), which were also shown in iBAT of *Mettl3*-BKO mice [5]. These data suggest that decreased m^6^A modification in *Prdm16* and *Pparg* transcripts leads to a reduction of PRDM16 and PPARγ, which may further cause the downregulation of BAT-selective genes such as *Ucp1*.

### BAT-specific overexpression of *Mettl3* ameliorates the impaired maturation of BAT in *Wtap*-BKO mice

**Figure 7:**
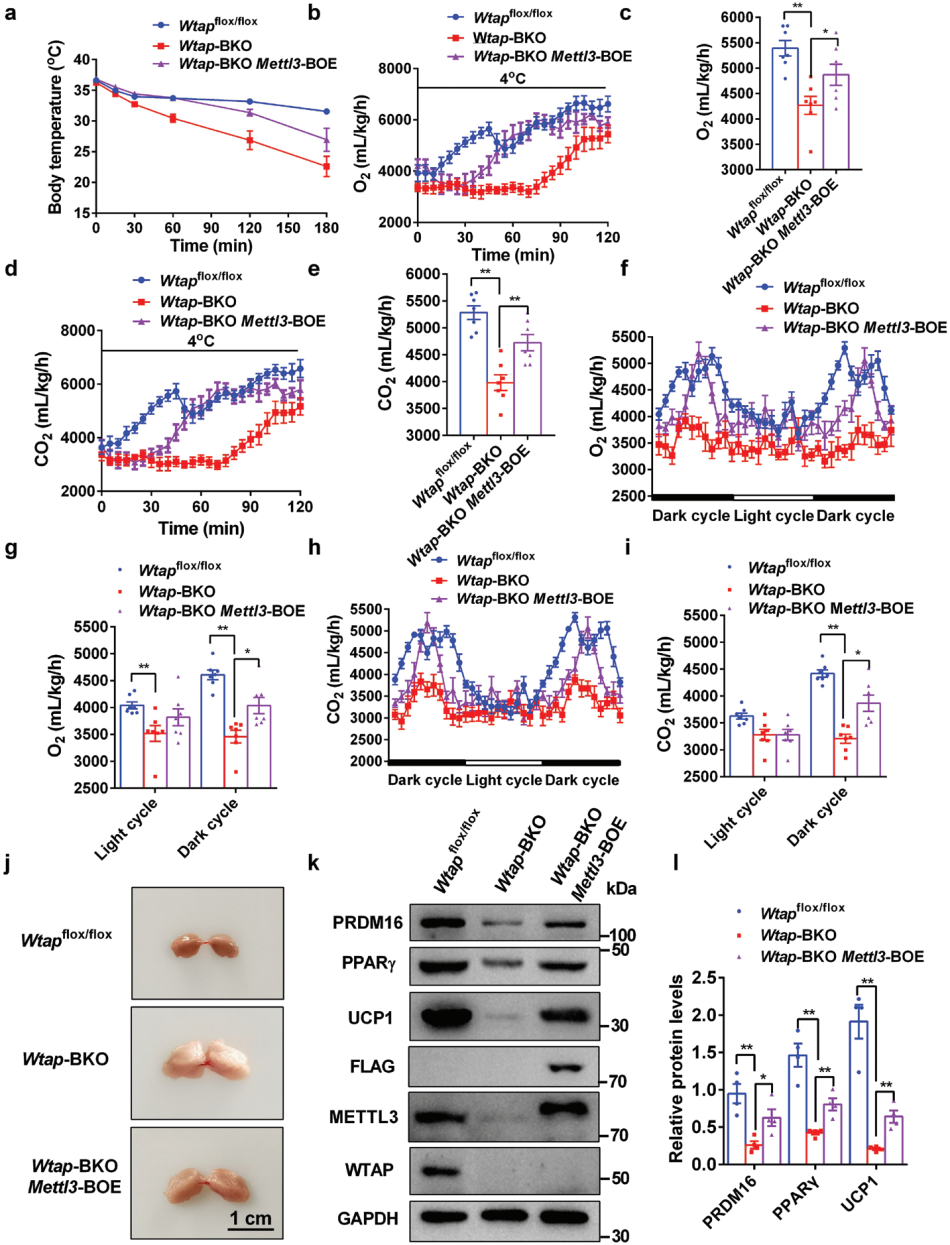
BAT-specific overexpression of *Mettl3* ameliorates the impaired maturation of BAT in *Wtap*-BKO mice. *STOP*-*Mettl3* transgenic mice were crossed with *Wtap*-BKO mice to generate *Wtap*-BKO/*Mettl3*-BOE mice. (a) The rectal temperature of 10-week-old *Wtap*^flox/flox^, *Wtap*-BKO, and *Wtap*-BKO/*Mettl3*-BOE mice during acute cold exposure (4°C) (*n* = 7). (b–e) The O_2_ consumption and CO_2_ production rates in 10-week-old *Wtap*^flox/flox^, *Wtap*-BKO, and *Wtap*-BKO/*Mettl3*-BOE mice during acute cold exposure (4°C) (*n* = 7). (f–i) The O_2_ consumption and CO_2_ production rates in 10-week-old *Wtap*^flox/flox^, *Wtap*-BKO, and *Wtap*-BKO/*Mettl3*-BOE mice at room temperature (*n* = 7). (j) Representative images of iBATs in *Wtap*^flox/flox^, *Wtap*-BKO, and *Wtap*-BKO/*Mettl3*-BOE mice at the age of 12 weeks. (k and l) PRDM16, PPARγ, UCP1, WTAP, Flag, METTL3, and GAPDH protein levels in iBATs of 12-week-old *Wtap*^flox/flox^, *Wtap*-BKO, and *Wtap*-BKO/*Mettl3*-BOE mice were determined by immunoblotting (*n* = 4) (k). Quantifications of the protein levels were from four independent mice per genotype (l). *P < 0.05. **P < 0.01. Data represent the mean ± SEM. *n* was the number of biologically independent mice.

BAT-specific deletion of *Wtap* resulted in impaired maturation of iBAT, which is likely due to the decreased METTL3-mediated m^6^A mRNA modification. To determine whether METTL3 is really involved in this process, we restored METTL3 in *Wtap*-BKO mice by crossing BAT-specific *Mettl3* overexpressing mice with *Wtap*-BKO mice. BAT-specific overexpression of *Mettl3* in *Wtap*-BKO mice rescued the impaired thermogenesis in *Wtap*-BKO mice, as revealed by higher body temperature (Fig. 7a) and higher O_2_ consumption (Fig. 7b and c) and CO_2_ production rates (Fig. 7d and e) during acute cold challenge. More importantly, at room temperature, BAT-specific overexpression of METTL3 in *Wtap*-BKO mice increased O_2_ consumption and CO_2_ production rates compared with *Wtap*-BKO (Fig. 7f–i), indicating that BAT-specific overexpression of *Mettl3* in *Wtap*-BKO mice rescued the impaired energy expenditure and thermogenesis in *Wtap*-BKO mice. BAT-specific overexpression of *Mettl3* in *Wtap*-BKO mice did not change the food intake but partially rescued the increased physical activity in *Wtap*-BKO mice at the light cycle (Supplementary Fig. S7a and b). BAT-specific overexpression of *Mettl3* in *Wtap*-BKO mice partially rescued the impaired development of iBAT in *Wtap*-BKO mice, as revealed by less whitening of iBAT (Fig. 7j), less steatotic hypertrophy (Supplementary Fig. S7c), and higher expression of PRDM16, PPARγ, and UCP1 (Fig. 7k and l). These data indicate that WTAP regulates postnatal development of BAT at least partially depending on METTL3.

## Discussion

Brown adipocyte maturation during postnatal development is essential for BAT to maintain energy homeostasis in rodents. However, the molecular mechanisms that determine the maturation of BAT during postnatal development are not fully understood. We recently demonstrated that METTL3-mediated m^6^A mRNA modification is essential for BAT development and energy expenditure [5]. In this study, we demonstrated that WTAP, an iBAT-enriched METTL3-binding protein, controls the postnatal development of iBAT and thermogenesis by regulating METTL3-mediated m^6^A mRNA modification.

WTAP shows a similar expression pattern and function in iBAT with METTL3. WTAP is also selectively expressed in iBAT and associated with the postnatal development of iBAT. Deletion of *Wtap* leads to dramatically impaired brown adipocyte differentiation by suppressing brown fat-related genes both *in vivo* and *in vitro*. *Wtap*-BKO mice display impaired postnatal development of iBAT and thermogenesis. iBAT in *Wtap*-BKO mice displays whitening at a very early age. Adult *Wtap*-BKO mice show reduced energy expenditure and CL 316,243-induced browning of WAT. All of these phenotypes in *Wtap*-BKO mice are due to the decreased expression of BAT-selective genes such as *Prdm16*, *Pparg*, *Pgc1a*, and *Ucp1* in the iBAT. Impaired maturation of brown adipocytes in *Wtap*-BKO mice leads to the dynamic changes of cell heterogeneity in iBAT. Macrophages and T/B lymphocytes are significantly increased in the iBAT of *Wtap*-BKO mice. *Prdm16*, *Pparg*, and *Ppargc1α* mRNA levels are significantly decreased in brown adipocytes, whereas fibrosis-related genes, such as *Col1a1* and *Col3a1*, are dramatically upregulated in fibroblasts and other cell types. Immatured brown adipocytes may secrete adipokines, which may recruit macrophages and T/B lymphocytes into iBAT. Inflammation and fibrosis may further lead to the impaired thermogenesis in *Wtap*-BKO mice. Interestingly, aging or BAT-specific deletion of *Prdm16* causes fibrosis, which leads to the decreased beige adipogenesis [30]. Therefore, downregulation of *Prdm16* is one of the molecular mechanisms in *Wtap*-BKO mice. However, BAT-specific *Prdm16* knockout mice show normal postnatal development of iBAT at a young age [4], whereas *Wtap*-BKO mice display severely impaired postnatal development of iBAT, which suggests that WTAP in iBAT has certain actions that probably do not occur solely through regulation of *Prdm16* expression.

Similar to the results observed in *Mettl3*-BKO mice, PPARγ and UCP1 protein levels are also significantly downregulated in *Wtap*-BKO mice, indicating that WTAP may regulate postnatal development of iBAT through modulation of METTL3. BAT-specific deletion of *Wtap* reduces METTL3 protein levels but not affect its mRNA levels. WTAP interacts with METTL3 and regulates its protein stability. Deletion of *Wtap* increases proteasome-mediated degradation of METTL3. BAT-specific deletion of *Wtap* decreases m^6^A modification in lots of transcripts, many of which are consistent with those in the iBAT of *Mettl3*-BKO mice. For example, the m^6^A modifications in *Prdm16* and *Pparg* transcripts are both decreased in the iBAT of *Wtap*-BKO and *Mettl3*-BKO mice. YTHDF2, one of the m^6^A reader proteins, likely recognizes these m^6^A modifications and regulates the expression of *Prdm16* and *Pparg* [5]. The m^6^A modification of *Ucp1* transcript does not change in the iBAT of *Wtap*-BKO mice, indicating that the downregulation of *Ucp1* is likely due to the downregulation of *Prdm16* and *Pparg*, but less likely due to its m^6^A modification. BAT-specific overexpression of METTL3 partially rescues the downregulation of PRDM16, PPARγ, and UCP1, which further rescues the impaired thermogenesis and energy expenditure in *Wtap*-BKO mice. These results indicate that WTAP regulates the postnatal development of iBAT and thermogenesis at least partially through METTL3. We notice that BAT-specific overexpression of METTL3 does not fully rescue the phenotypes observed in *Wtap*-BKO mice, indicating that other molecular mechanisms may also contribute to WTAP function in BAT. Recently, it has been shown that WTAP and METTL3 can also bind to gene promoters and regulate chromatin accessibility [14, 22]. It is necessary to check whether WTAP and METTL3 can directly regulate gene transcription in iBAT in the future.

To our surprise, normal food intake, reduced energy expenditure, and impaired browning of WAT do not promote HFD-induced obesity in *Wtap*-BKO mice, which is likely due to the increased physical activity. BAT-specific deletion of *Wtap* increases physical activity during the daytime, whereas BAT-specific overexpression of METTL3 partially rescues this phenotype. However, BAT-specific deletion of *Mettl3* does not change physical activity [5]. These results indicate that WTAP in BAT regulates physical activity independent of METTL3. Although the Cre recombinase in *Ucp1*-Cre transgenic mice can be detected in the brain [24], the *Ucp1*-iCre mice we used do not show Cre expression in the brain. Consistently, *Ucp1*-iCre mediated deletion of *Wtap* is specific in the iBAT but not brain. These results rule out the possibility that nonspecific deletion of *Wtap* in brain or hypothalamus leads to the increased physical activity in *Wtap*-BKO mice. Instead, *Wtap*-BKO mice show increased running time likely due to the increased skeletal muscle function. It is possible that BAT-specific deletion of *Wtap* changes the expression and secretion of certain adipokines that may further target skeletal muscle or brain and then regulate physical activity. This hypothesis needs further investigation.

WTAP and METTL3 as m^6^A writer proteins promote postnatal development of iBAT, thermogenesis, and browning of white adipocytes. Fat mass and obesity-associated protein (FTO) as an m^6^A eraser protein plays an opposite role in the browning of white adipocytes and energy expenditure. FTO deficiency has been shown to promote the browning of white adipocytes by inducing UCP1 expression in white adipocyte [31, 32]. Some m^6^A reader proteins, such as YTHDF1-3 and YTHDC1-2, may regulate the postnatal development of iBAT, thermogenesis, and browning of white adipocytes [5, 32], which needs further study.

In conclusion, we have shown that WTAP is an essential regulator of the postnatal development of iBAT and thermogenesis. BAT-specific deletion of *Wtap* severely impairs maturation of BAT by decreasing the expression of BAT-selective genes. WTAP deficiency decreases m^6^A mRNA modification by reducing the protein stability of METTL3. BAT-specific overexpression of *Mettl3* partially rescues the phenotypes in BAT of *Wtap*-BKO mice. These data demonstrate that WTAP plays an essential role in iBAT postnatal development and thermogenesis by stabilizing METTL3.

## Methods

### Animal experiments

Mice were housed on a 12-h light/12-h dark cycle and fed a normal chow with free access to water. For diet-induced obesity studies, mice were fed with an HFD (PD6001, 60% fat, Changzhou SYSE Bio-Tec. Co., Ltd.). *Wtap*^flox/flox^ mice, in which the exon 4 of *Wtap* gene was flanked by two loxp sites, were generated by using CRISPR-Cas9 technique (Supplementary Fig. S1a). *Ucp1*-iCre mice, in which IRES-Cre was inserted between exon 6 and the 3ʹ-UTR to allow *Ucp1* and iCRE expression at the same time with lower levels, have been shown previously [5, 25]. *Wtap*-BKO mice were generated by crossing *Wtap*^flox/flox^ mice with *Ucp1*-iCre mice. The chemicals, reagents, and antibodies used in this paper were listed in the Supplementary Table S3.

### Global m^6^A measurement

Global m^6^A levels in total RNA were quantified by the EpiQuik m^6^A RNA Methylation Quantification Kit (P-9005, Epigentek) following manufacturers’ specifications and using 200 ng input.

### Food intake, physical activity, and energy expenditure measurement

For metabolic studies, mice were housed individually in metabolic cages (Promethion, Sable Systems, LasVegas, NV), and had free access to food and water. O_2_ consumption and CO_2_ production rates were monitored for 72 h. Food intake and physical activity were measured simultaneously with metabolic measurements.

### Glucose tolerance test and insulin tolerance test

For the glucose tolerance test experiment, mice fasted for 6 h were injected intraperitoneally with D-glucose (1 g/kg). For the insulin tolerance test experiment, mice fasted for 6 h were injected intraperitoneally with human insulin (Lily) (1 U/kg). Blood glucose levels were measured from the tail vein at indicated time using a glucometer as described previously [33].

### *In vivo* insulin stimulation assay

Twenty-one-week-old HFD-fed *Wtap*^flox/flox^ and *Wtap*-BKO mice were fasted for 20–24 h, anesthetized, and administrated insulin (2 U/kg body weight) via inferior vena for 5 min. Livers were isolated and homogenized in a lysis buffer (50 mmol/L Tris HCl, pH 7.5, 1.0% NP-40, 150 mmol/L NaCl, 2 mmol/L EGTA, 1 mmol/L Na_3_VO_4_, 100 mmol/L NaF, 10 mmol/L Na_4_P_2_O_7_, 1 mmol/L phenylmethylsulfonyl fluoride (PMSF), 10 mg/mL aprotinin, and 10 mg/mL leupeptin). Liver extracts were immunoblotted with antibodies against phospho-AKT (pSer473) and AKT. The detailed information for antibodies is listed in Supplementary Table S3.

### Cold-stress experiment

For cold exposure experiment, an individual mouse was placed in a single cage in a cold room (4°C) with free access to water. The core body temperature was monitored using a rectal probe (7001HT, Phyritemp) at each time point.

### Chronic CL 316,243 treatment

Chronic CL 316,243 treatment was followed a published method [5]. Briefly, *Wtap*^flox/flox^ and *Wtap*-BKO mice were injected with CL 316,243 at 1 mg/kg body weight or an equal volume of saline daily for 4 days. Mice were sacrificed on day 5 without additional injection.

### Immunoblotting

Cells or tissues were homogenized in an L-RIPA lysis buffer. Protein was separated by SDS-PAGE, immunoblotted with the indicated antibodies, and visualized using the ECL. The information for antibodies is listed in Supplementary Table S3.

### Quantitative real-time PCR (qPCR)

Total RNA isolation and RT-qPCR were performed as shown before [33, 34]. RNA abundance was measured using Absolute qRT-PCR SYBR Mix (Roche, Mannheim, Germany) and Roche LightCycler 480 RT PCR system (Roche, Mannheim, Germany). The expression of individual gene was normalized to the expression of 36B4, a housekeeping gene. Primers for real-time qRT-PCR were listed below: *Mettl3*-F: CTTGCCATCTCTACGCCAGA, *Mettl3*-R: TCATGGCAGACAGCTTGGAG; *Wtap*-F: CTTCCGCGGACTGTCTCC, *Wtap*-R: GGTCATCTTGCACCCCGAG; 36B4-F: AAGCGCGTCCTGGCATTGTCT, 36B4-R: CCGCAGGGGCAGCAGTGGT.

### Primary brown adipocyte culture and adenovirus infection

Isolation of brown fat SVF and differentiation of primary brown preadipocytes were performed as described previously [5, 35, 36]. In brief, the interscapular brown fat pad was dissected from 4- to 6-week-old *Wtap*^flox/flox^ mice, minced, and then digested for 20–30 min at 37°C in PBS containing 10 mmol/L CaCl_2_, 1.5 mg/mL Collagenase type II, and 1.4 U/mL Dispase II. Digested tissues were filtered through a 40 μm cell strainer to remove large pieces and then centrifuged for 10 min at 1000 g to pellet the SVF cells. SVF cells were resuspended in a complete culture medium (DMEM with 10% FBS and Pen/Strep) and then plated on collagen-coated 24-well plates. For preadipocyte differentiation, cells grown to 100% confluence (Day 0) were exposed to induction in DMEM containing 2 μg/mL dexamethasone, 1 μmol/L insulin, 0.5 mmol/L isobutylmethylxanthine, 1 μmol/L rosiglitazone, 1 nmol/L T3, 62.5 μmol/L indomethacin, and 10% FBS. Three days after induction (from Day 3), cells were maintained in media containing 1 μmol/L insulin, 1 nmol/L T3, and 10% FBS until ready for harvest (generally day 6–7 after differentiation). All chemicals for cell culture were obtained from Sigma-Aldrich. For adenoviral infection of primary SVF cells, 100% confluent cells were infected with Cyclization Recombination Enzyme (Cre)- or βGal-expressing adenovirus in a growth medium overnight. For western blot analysis and Oil red O staining, the infected cells were switched to an induction medium for 72 h to induce adipogenic differentiation and then maintained in a differentiation medium for 4 days. Cells were then used for western blot analysis and Oil red O staining. For protein stability assay, the infected cells were switched to an induction medium for 24 h to induce adipogenic differentiation and then were treated with cycloheximide (CHX, 50 ng/mL) for indicated periods. Cells were then harvested for immunoblotting analysis of METTL3 and GAPDH protein levels. The relative METTL3 protein levels were represented as the percentage of the band densities at 0 h.

### *Ex vivo* experiments

The iBAT was dissected from 4- to 6-week-old *Wtap*^flox/flox^ and *Wtap*-BKO mice and cut into 10 mg pieces. Pieces of iBAT were randomly divided into two groups. One group was treated with or without MG132 (100 μmol/L) and the other group was treated with or without leupetin (100 μmol/L) at 37°C for 6 h. METTL3 and GAPDH protein levels were measured by immunoblotting.

### Exercise capacity measurement

*Wtap*^flox/flox^ and *Wtap*-BKO mice at the age of 8 weeks were trained once a day for 3 days on a treadmill (ZH-PT, ANHUI ZHENGHUA BIOLOGIC APPARTUS FACILITIES CO., LTD) at 10 m/min for 30 min. For exercise capacity measurement, the treadmill was set up an initial speed of 10 m/min for 30 min, the speed was increased by 2 m/min every 20 min until the mice were exhausted (mice spent more than 5 s on the electric shocker without resuming running).

### RNA-seq, m^6^ARIP-seq, and snRNA-seq

RNA-seq was performed as described previously [5, 34]. Briefly, total RNA was extracted using Tripure Isolation Reagent (Roche, Mannheim, Germany) from iBAT of *Wtap*^flox/flox^ and *Wtap*-BKO mice at the age of 8 weeks old (*n* = 3 for each group). RNA-seq was performed by using Illumina NovaSeq 6000 platform. About 150 bp paired-end clean reads were aligned to the mouse reference genome (Ensemble_GRCm38.p6) with Hisat2 (version 2.0.5), and the aligned reads were used to quantify mRNA expression by using featureCounts (version 1.5.0-p3). Differential expression analysis of two groups (three biological replicates per group) was performed using the DESeq2 R package (1.16.1). DESeq2 provides statistical routines for determining differential expression in digital gene expression data using a model based on the negative binomial distribution. The resulting *P* values were adjusted using the Benjamini and Hochberg’s approach for controlling the false discovery rate. Genes with an adjusted *P*-value <0.05 found by DESeq2 were assigned as differentially expressed. GO enrichment analysis of differentially expressed genes was implemented by the clusterProfiler R package, in which gene length bias was corrected. GO terms with corrected *P* value less than 0.05 were considered significantly enriched by differential expressed genes.

m^6^ARIP-seq was performed as described previously with modifications [37]. Briefly, total RNA was extracted using Tripure Isolation Reagent (Roche, Mannheim, Germany) from iBAT of *Wtap*^flox/flox^ and *Wtap*-BKO mice at the age of 8 weeks old. Each sample (300 μg total RNA) was pooled from 8 mice for each group. Poly(A)^+^ RNA was purified using Dynabeads™ mRNA Purification Kit (Invitrogen) following the manufacturer’s instructions. Chemically fragmented poly(A)^+^ RNA was incubated with m^6^A antibody (202003, Synaptic System) for immunoprecipitation following the standard protocol of Magna MeRIP^TM^ m^6^A Kit (17-10499, MERCK). Enrichment of m^6^A mRNA was then analyzed by high-throughput sequencing using Illumina NovaSeq 6000 platform. The m^6^A peaks were detected by MACS2, and the motif search was detected by HOMER as shown before [38].

Single nucleus RNA-seq was performed following published methods [39–41]. iBAT tissues were harvested from *Wtap*^flox/flox^ and *Wtap*-BKO mice at the age of 8 weeks quickly, frozen in liquid nitrogen, and stored at −80°C until use. Nuclei were isolated from frozen iBAT samples for 10× snRNA-seq. Each sample was pooled from 4 mice for each group. All sample handing steps were performed on ice. Frozen iBAT tissues were dounced in 3 mL of lysis buffer (10 mmol/L Tris (pH 7.4), 10 mmol/L NaCl, 3 mmol/L MgCl_2_, 0.05% (v/v) NP-40 detergent, and 1 U/μL RNase inhibitor). The samples were incubated in a total of 5 mL of lysis buffer for 5 min. The samples were passed through a 30-μm cell strainer and then spun for 5 min at 500 × g. The nuclei were resuspended in 5 mL wash buffer (10 mmol/L Tris (pH7.4), 10 mmol/L NaCl, 3 mmol/L MgCl_2_, 1% BSA, 1 mmol/L DTT, and 1 U/μL RNase inhibitor) by pipetting up and down 8 times and washed for 3 times. The nuclei were resuspended in 1 mL wash buffer, mixed with 25% Optiprep, layered on a 29% Optiprep cushion, and spun for 30 min at 10,000 × g. Nuclei were resuspended in wash buffer and washed 3 times. An aliquot of nuclei from each sample was stained with AO/PI, and counted in a hemocytometer. The nuclei were resuspended in Nuclei Resuspension Buffer (Nuclei buffer (10× Genomics, 20×) 1×, 1mmol/L DTT, and 1 U/μL RNase inhibitor) to achieve a concentration of ~1 × 10^6^ nuclei per mL. The nuclei were then immediately loaded on the 10x Chromium controller (10× Genomics) according to the manufacturer’s protocol. Briefly, the nuclei suspension was loaded into Chromium microfluidic chips with 30 v3.1 chemistry and barcoded with a 10× Chromium Controller (10× Genomics). RNA from the barcoded cells was subsequently reverse-transcribed, and sequencing libraries were constructed with reagents from a Chromium Single Cell 30 v3.1 reagent kit (10× Genomics) according to the manufacturer’s instructions. Sequencing was performed with Illumina NovaSeq 6000 according to the manufacturer’s instructions (Illumina). Analyses were conducted using the Seurat program (Seurat v4.0). For quality control, we removed cells for which fewer than 500 UMIs. Simultaneous data normalization/scaling and variable feature detection were performed using “LogNormalize”. Cells were integrated by canonical correlation analysis. Cells were clustered via Seurat’s shared nearest neighbor clustering algorithm (“FindNeighbors” and “FindClusters”) using the top 20 PCs and a resolution of 0.5. The top eight most variable PCs were used for subsequent clustering. The clustering was visualized using t-Distributed Stochastic Neighbor Embedding (tSNE). Cluster markers were obtained with the Seurat function “FindAllMarkers” using default settings.

### Tissue metabolite extraction

A tissue sample was homogenized at −20°C for 30 min. Methanol:water (*v:v*, 80:20) was prechilled at −80°C overnight, and 3 mL was added to the tissue sample homogenate. The homogenate was then incubated at −80°C for 30 min and decanted to a 15 mL centrifuge tube. The supernatant was then collected in another 15 mL centrifuge tube after a 4000 × g, 10 min centrifuge at 4°C. The 80% methanol extracted metabolites were then dried using a SpeedVac (LABCONCO Refrigerated CentriVap Concentrator) and stored at −80°C before mass spectrometry analysis.

### Targeted metabolomic analysis

The metabolomic approach was adopted from a published method [42]. In general, samples were resuspended in 50 μL of water:acetonitrile (*v:v*, 50:50), and 5 μL was injected into a 6500QTRAP mass spectrometer (SCIEX) coupled to an HPLC system (Shimadzu). Metabolites were eluted via hydrophilic interaction chromatography (HILIC) using a 4.6 mm i.d. × 10 cm AmideXBridge column (Waters) with a flow rate of 400 μL/min using buffer A (20 mmol/L ammonium hydroxide/20 mmol/L ammonium acetate (pH 9.2) at a 95:5 ratio with water:acetonitrile) and buffer B (acetonitrile). Gradients were run from 85% buffer B to 42% buffer B at 0–5 min, from 42% buffer B to 0% buffer B at 5–16 min, 0% buffer B was held from 16–24 min, from 0% buffer B to 85% buffer B at 24–25 min, and 85% buffer B was held for another 7 min. All ions were acquired by selected reaction monitoring transitions in a positive and negative mode switching fashion. Electrospray ionization (ESI) voltage was +4900 and −4500 V in positive or negative mode, respectively.

### Statistical analysis

Data were presented as means ± SEM. Differences between groups were analyzed by Student’s *t* tests. *P* < 0.05 was considered statistically significant, *P < 0.05. **P < 0.01.

## Supplementary Material

loac028_suppl_Supplementary_Material

loac028_suppl_Supplementary_Table_S1

loac028_suppl_Supplementary_Table_S2

## Data Availability

The data supporting the findings are available within the article and Supplementary Information. RNA-seq data files have been deposited into Gene Expression Omnibusdatabase with accession number GSE202510. m6ARIP-seq data that support the findings of this study have been deposited in GEO under accession code GSE202668. snRNA-seq data that support the findings of this study have been deposited in GEO under accession code GSE202630.
