# Supplementary material for: WTAP regulates postnatal development of brown adipose tissue by stabilizing METTL3 in mice
Source: Life Metab. 2022 Oct 10;1(3):270–84. doi: 10.1093/lifemeta/loac028 (PMC11749075; doi:10.1093/lifemeta/loac028)
Supplement: loac028_suppl_Supplementary_Material [file loac028_suppl_Supplementary_Material.pdf]

Supplementary Information

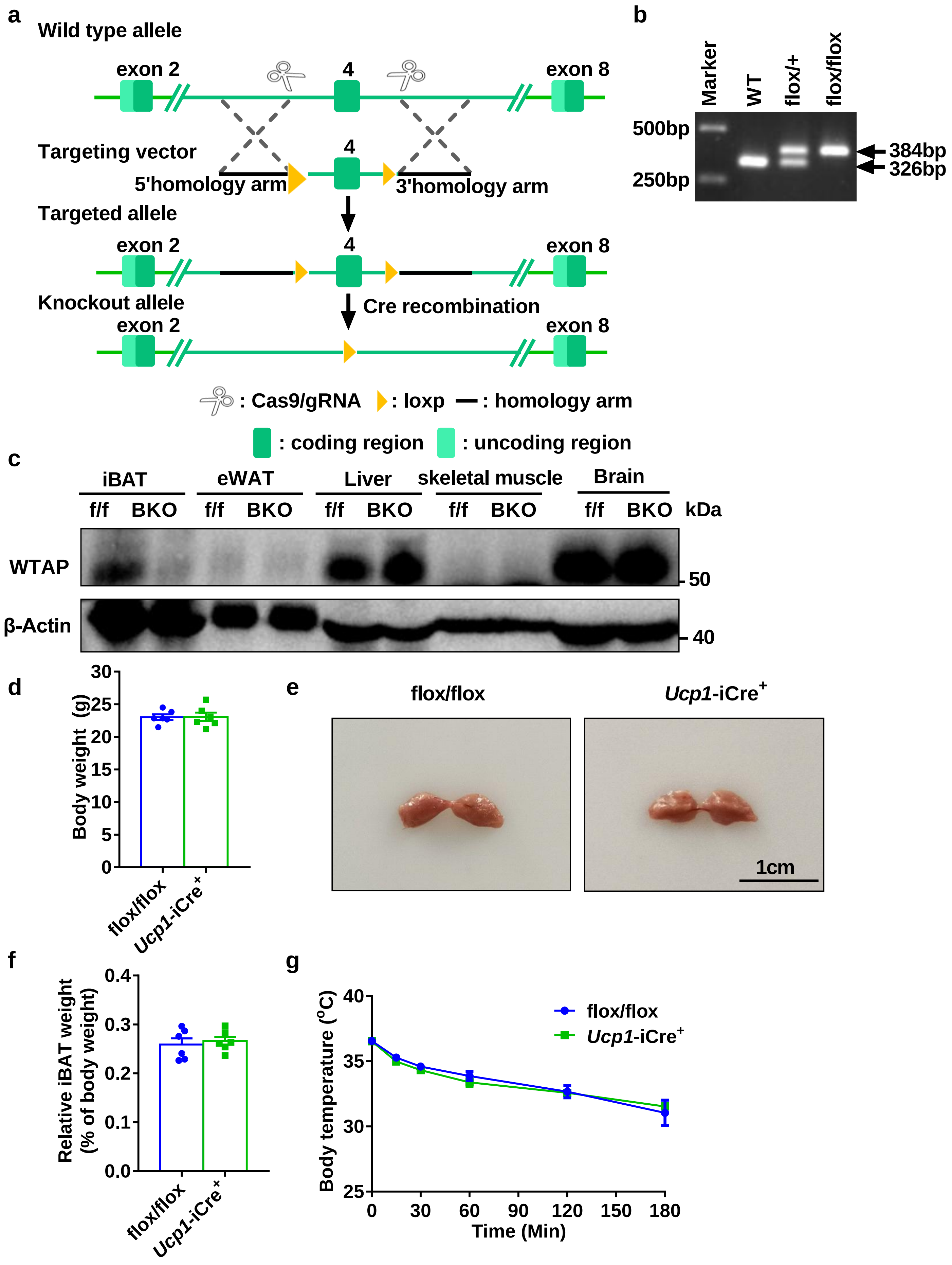
a
Wild type allele
exon 2
4
exon 8
Targeting vector
4
5'homology arm
3'homology arm
Targeted allele
exon 2
4
exon 8
Knockout allele
Cre recombination
exon 2
exon 8
: Cas9/gRNA
: loxp
: homology arm
: coding region
: uncoding region
b
Marker
WT
flox/+
flox/flox
500bp
250bp
384bp
326bp
c
iBAT
eWAT
Liver
skeletal muscle
Brain
f/f BKO f/f BKO f/f BKO f/f BKO f/f BKO kDa
WTAP
β-Actin
50
40
d
Body weight (g)
30
25
20
15
10
5
0
flox/flox
Ucp1-iCre+
e
flox/flox
Ucp1-iCre+
1cm
f
Relative iBAT weight (% of body weight)
0.4
0.3
0.2
0.1
0.0
flox/flox
Ucp1-iCre+
g
Body temperature (°C)
40
35
30
25
0 30 60 90 120 150 180
Time (Min)
flox/flox
Ucp1-iCre+

Supplementary Figure 1. Generation of *Wtap*$^{\mathbf{flox/flox}}$ and *Wtap*-BKO mice.

(a) Generation of *Wtap*$^{flox/flox}$ mice. *Wtap* alleles and targeting construct were shown. Top: wildtype *Wtap* wt allele (exons indicated as solid green rectangles). Middle: targeting vector containing two loxP sites (yellow triangles). Insertion of the targeting vector by CRISPR/Cas9 technique yielded the *Wtap* lox allele.

(b) Genotyping of WT, *Wtap*$^{flox/-}$ and *Wtap*$^{flox/flox}$ mice.

(c) WTAP and β-Actin protein levels in iBAT, eWAT, liver, skeletal muscle and brain of 8-week-old *Wtap*$^{flox/flox}$ and *Wtap*-BKO mice were measured by immunoblotting.

(d) The body weight of 8-week-old *Wtap*$^{flox/flox}$ and *Ucp1*-iCre mice (n=6)**.**

(e, f) Gross appearance and relative weight of iBATs in *Wtap*$^{flox/flox}$ and *Ucp1*-iCre mice at 8-week old (n=6).

(g) The rectal temperature of 8-week-old *Wtap*$^{flox/flox}$ and *Ucp1*-iCre mice during acute cold exposure (4℃) (n=6).

Data represent the mean ± SEM. n was the number of biologically independent mice.

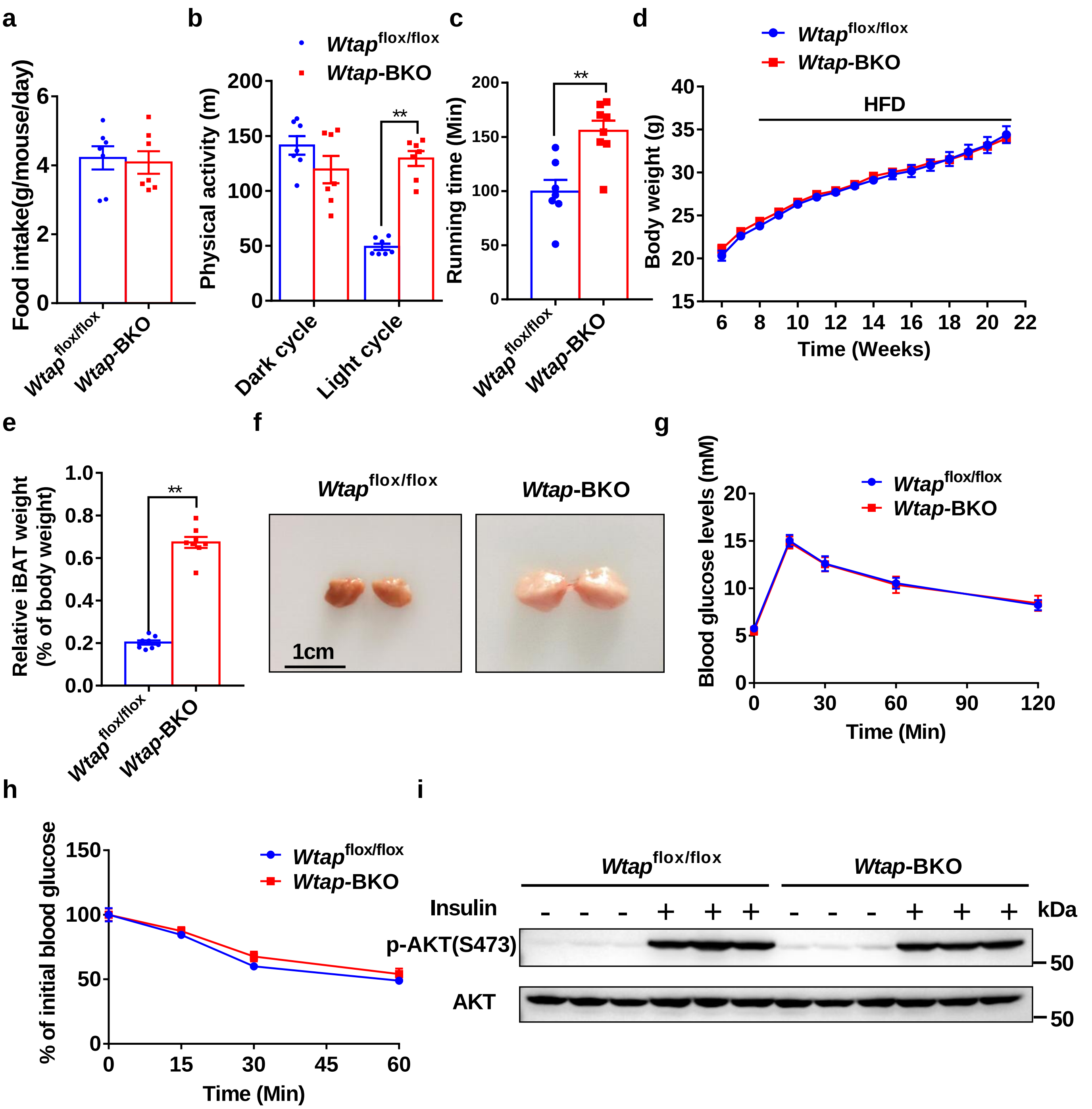

Supplementary Figure 2. **BAT-specific knockout of *Wtap* increases physical activity during light time and does not promote HFD-induced obesity.**

(a, b) The food intake and physical activity of 10-week-old *Wtap*$^{flox/flox}$ and *Wtap*-BKO mice (n=7).

(c) Running time of *Wtap*$^{flox/flox}$ and *Wtap*-BKO mice in a treadmill experiment (n = 7-8).

(d) The growth curve of *Wtap*$^{flox/flox}$ and *Wtap*-BKO mice fed HFD (n = 11).

(e-f) The relative iBAT weight and representative images of iBAT in 21-week-old HFD-fed *Wtap*$^{flox/flox}$ and *Wtap*-BKO mice (n=8).

(g, h) GTTs and ITTs of 21-week-old HFD-fed mice (n = 11).

(i) p-AKT(S473) and AKT protein levels of 21-week-old HFD-fed mice (n=3).

**, p< 0.01. Data represent the mean ±SEM. n was the number of biologically independent mice.

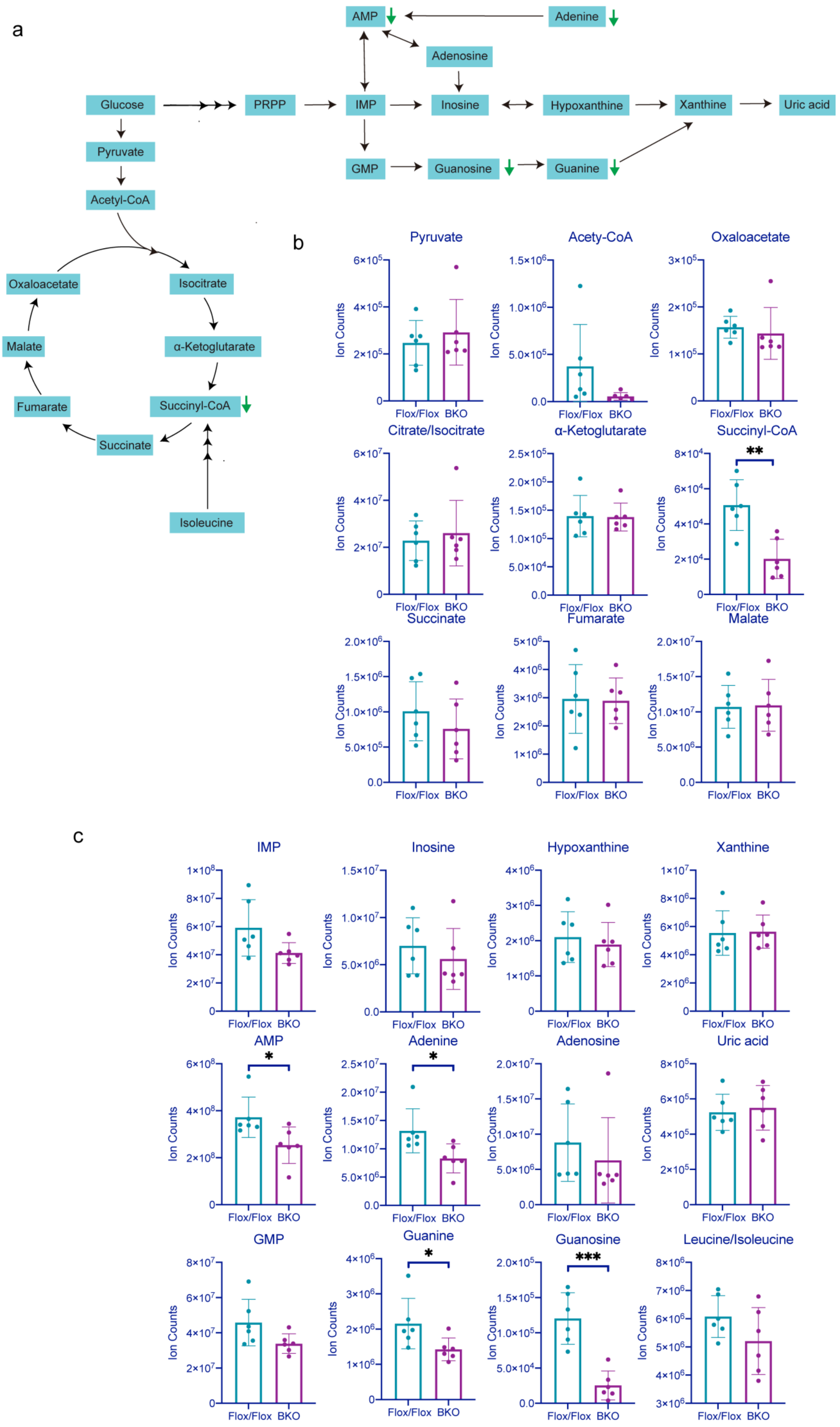
a
AMP
Adenine
Adenosine
Glucose
PRPP
IMP
Inosine
Hypoxanthine
Xanthine
Uric acid
GMP
Guanosine
Guanine
Pyruvate
Acetyl-CoA
Oxaloacetate
Isocitrate
Malate
α-Ketoglutarate
Fumarate
Succinyl-CoA
Succinate
Isoleucine
b
Pyruvate
Acety-CoA
Oxaloacetate
Citrate/Isocitrate
α-Ketoglutarate
Succinyl-CoA
Succinate
Fumarate
Malate
Ion Counts
Flox/Flox BKO
c
IMP
Inosine
Hypoxanthine
Xanthine
AMP
Adenine
Adenosine
Uric acid
GMP
Guanine
Guanosine
Leucine/Isoleucine

Supplementary Figure 3. Metabolic alteration in iBAT of *Wtap*-BKO mice

(a) The metabolic pathway of electron transport chain and purine de novo synthesis. (b) Metabolites involved in TCA cycle were measured in iBAT of *Wtap*$^{flox/flox}$ and *Wtap*-BKO mice at 8 weeks old. (c) The downregulated AMP and guanosine indicate that the purine de novo synthesis via pentose phosphate pathway is decreased. *, $p < 0.05$. **, $p < 0.01$. Data represent the mean ± SEM. n was the number of biologically independent mice.

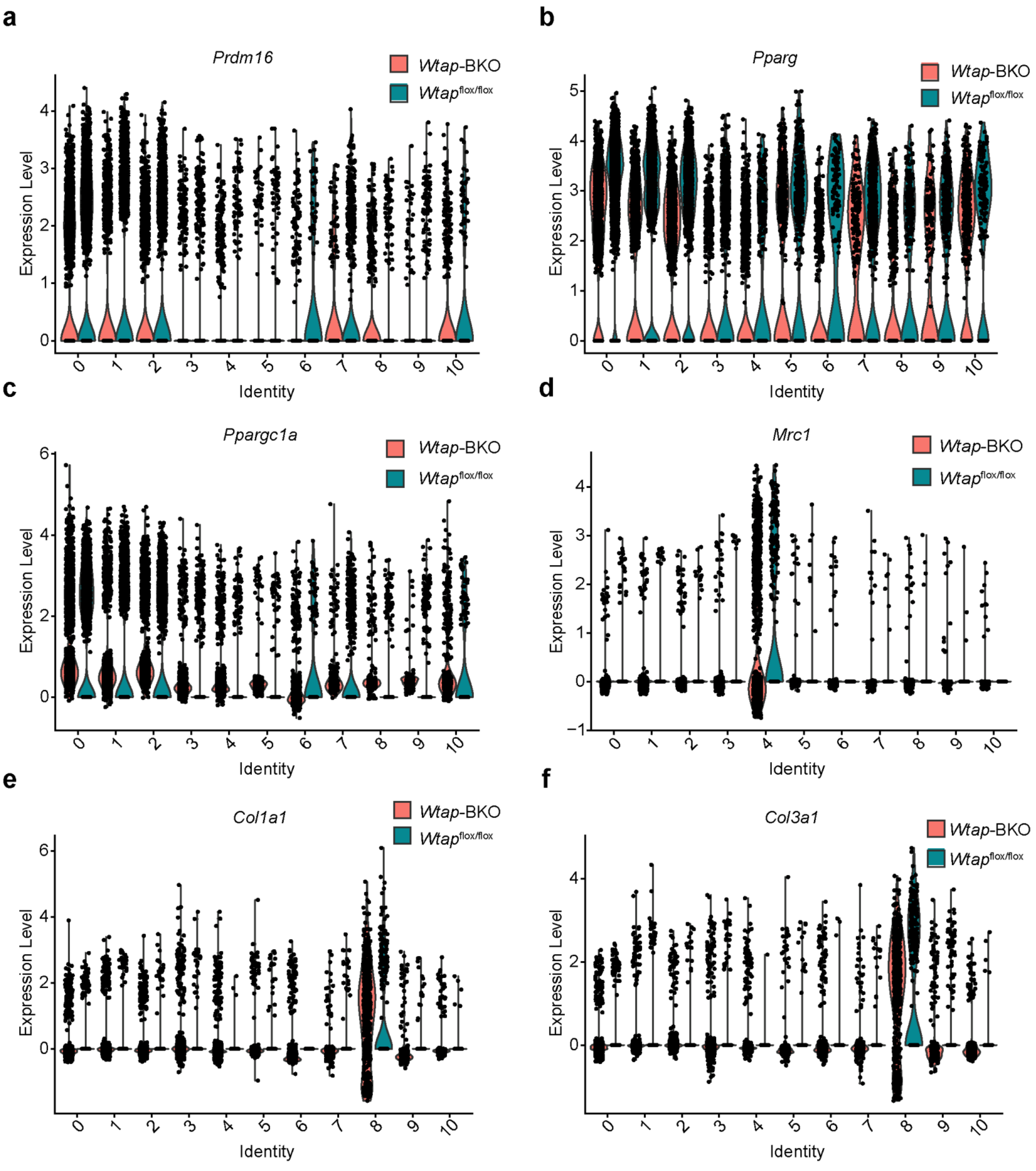

Supplementary Figure 4. BAT-specific deletion of *Wtap* largely changes cell heterogeneity in iBAT

(a-f) Cell nuclei were isolated from iBAT in *Wtap*$^{flox/flox}$ and *Wtap*-BKO mice at 8 weeks old. snRNA-seq analysis was performed. Violin plots showing the distribution of normalized expression values of *Prdm16* (a),

Pparg (b), *Ppargc1α* (c), *Mrc1* (d), *Col1a1*(e) and *Col3a1* (f) across cells that belong to the 11 cell clusters.

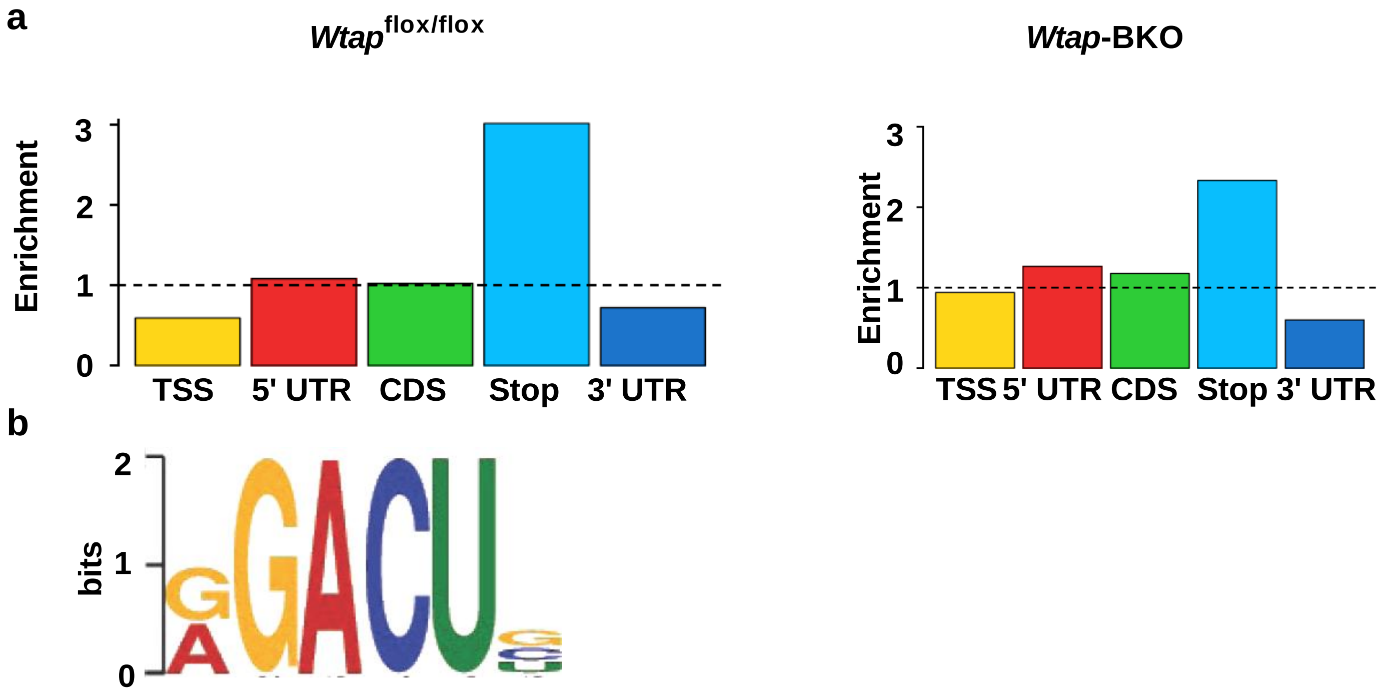

Supplementary Figure 5. WTAP is essential for m^6A mRNA modification in iBAT.

The MeRIP-seq analysis of iBATs were performed in 8-week-old *Wtap*$^{flox/flox}$ and *Wtap*-BKO mice. (a) The enrichment of m^6A-RIP-seq peaks in iBAT of 8-week-old *Wtap*$^{flox/flox}$ and *Wtap*-BKO mice. (b) Consensus motif of m^6A sites in iBAT of 8-week-old *Wtap*$^{flox/flox}$ mice (p=1.2e-814).

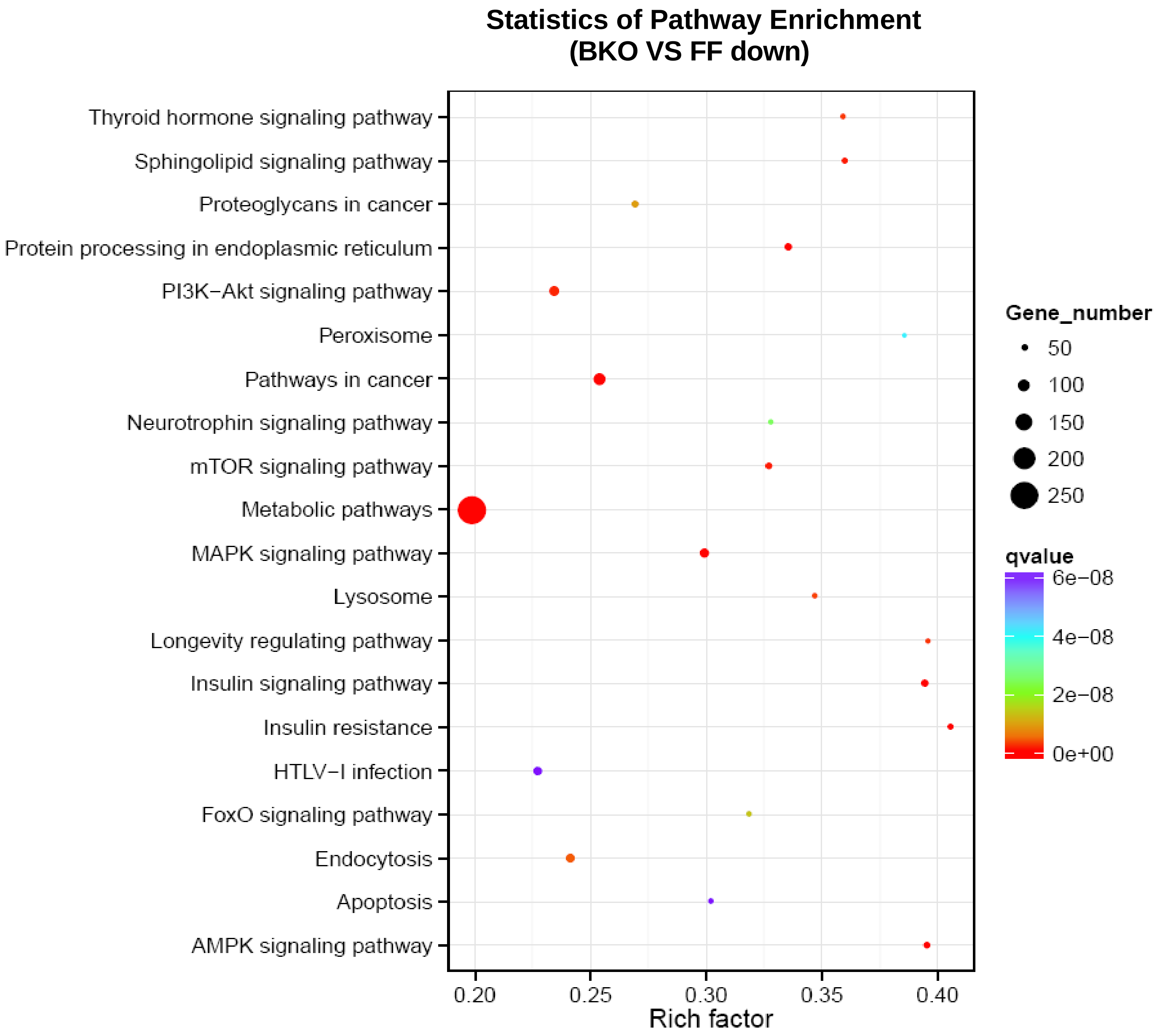

Supplementary Figure 6. Genes with downregulated m^{6}A peaks are associated with signaling pathways. The MeRIP-seq analysis of iBATs were performed in 8-week-old *Wtap*$^{flox/flox}$ and *Wtap*-BKO mice. KEGG analysis of the genes with downregulated m^{6}A peaks.

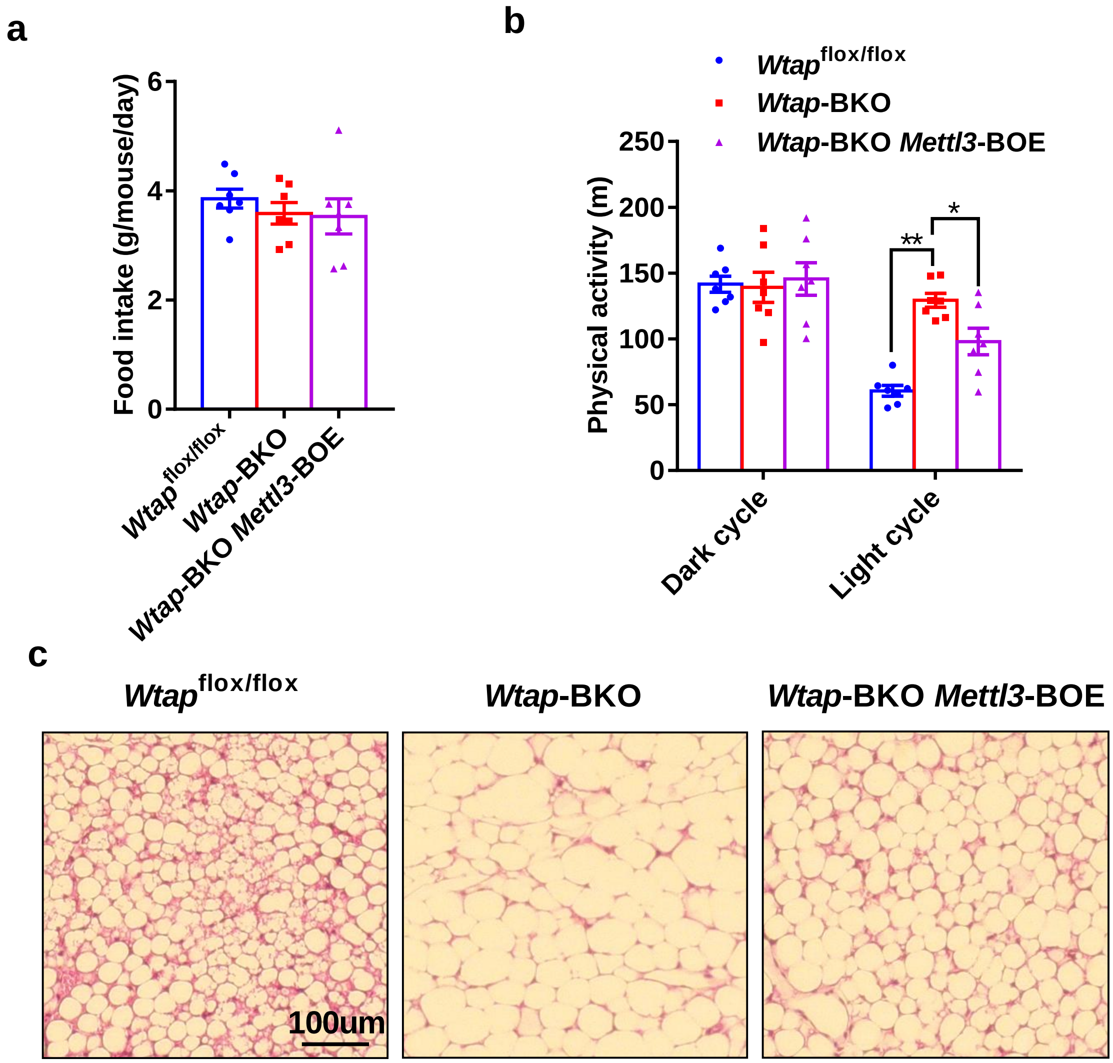

Supplementary Figure 7. BAT-specific overexpression of *Mettl3* in *Wtap*-BKO mice does not change the food intake but partially rescues the increased physical activity in *Wtap*-BKO mice at light time

(a, b) The food intake and physical activity of 10-week-old *Wtap*$^{flox/flox}$, *Wtap*-BKO and *Wtap*-BKO/*Mettl3*-BOE mice were measured (n=7 for each group). (c) H&E staining of iBATs in 10-week-old *Wtap*$^{flox/flox}$, *Wtap*-BKO and *Wtap*-BKO/*Mettl3*-BOE mice. *, p< 0.05. **, p< 0.01. Data represent the mean ± SEM. n was the number of biologically independent mice.

Supplementary Table 3

REAGENT or RESOURCE	SOURCE	IDENTIFIER
Antibodies		
METTL3	CST	Cat#96391
WTAP	proteintech	**Cat#** 10200-1-AP
UCP1	SIGMA	Cat#U6382
PGC-1α	proteintech	Cat#66369-1-lg
β-Actin	proteintech	Cat#60008-1-lg
PPARγ	proteintech	Cat#16643-1-AP
PRDM16	Abclonal	Cat# A11581
Total OXPHOS	abcom	Cat#ab110413
GAPDH	proteintech	Cat#60004-1-lg
Flag	SIGMA	Cat# F1804
AKT	CST	Cat#9272
P-AKT(S473)	CST	Cat#9271

Chemicals		
IBMX(isobutylmethylx anthine)	SIGMA	Cat#I5879
T3	SIGMA	Cat#T2877
ROSIGLITAZONE	SIGMA	Cat#R2408
INDOMETHACIN CRYSTALLINE	SIGMA	Cat#I7378
DEXAMETHASINE CRYSTALLINE	SIGMA	Cat#D1756

INSULIN	SIGMA	Cat#I5500
RIPA Buffer	Solarbio	Cat#R0020
Blue Plus II Protein Marker	Transgen Biotech	Cat#L50824
High Pure dNTPs	Transgen Biotech	Cat#20201025
GoTaq Green Master Mix	Promega	Cat#M7122
M-MLV reverse transcriptase	Promega	Cat#M1701
Random Primers	Promega	Cat#C1181
FBS	TRINTY TEK	Cat#01010102
Collagenase Type2	Worthington	Cat#LS004176
SYBR™ Green Mix	Roche	Cat#4913914001
TriPure Isolation Reagent	Roche	Cat#94015120
Methanol	Fisher Chemical	Cat#A452
Water	Fisher Chemical	Cat#A4
Acetonitrile	Fisher Chemical	Cat#A998
Ammonium acetate	Fisher Chemical	Cat#A114
Ammonium hydroxide	Fisher Chemical	Cat#470
MG132	Sigma	Cat#474787
leupetin	Sigma	Cat# L2884

Software		
Graphpad	Graphpad softwave	Graphpad.com
